# Mutual induced fit transition structure stabilization of corannulene's bowl-to-bowl inversion in a perylene bisimide cyclophane†

**DOI:** 10.1039/d3sc05341e

**Published:** 2023-11-27

**Authors:** Manuel Weh, Asja A. Kroeger, Olga Anhalt, Amir Karton, Frank Würthner

**Affiliations:** a Institut für Organische Chemie, Universität Würzburg Am Hubland 97074 Würzburg Germany wuerthner@uni-wuerzburg.de; b School of Molecular Sciences, The University of Western Australia 35 Stirling Highway Crawley WA 6009 Australia; c Research School of Chemistry, Australian National University Canberra ACT 2601 Australia; d Institute for Nanoscale Science & Technology, Flinders University Adelaide South Australia 5042 Australia; e Center for Nanosystems Chemistry, Bavarian Polymer Institute, Universität Würzburg Theodor-Boveri-Weg 97074 Würzburg Germany; f School of Science and Technology, University of New England Armidale NSW 2351 Australia amir.karton@une.edu.au

## Abstract

Corannulene is known to undergo a fast bowl-to-bowl inversion at r.t. *via* a planar transition structure (TS). Herein we present the catalysis of this process within a perylene bisimide (PBI) cyclophane composed of chirally twisted, non-planar chromophores, linked by *para*-xylylene spacers. Variable temperature NMR studies reveal that the bowl-to-bowl inversion is significantly accelerated within the cyclophane template despite the structural non-complementarity between the binding site of the host and the TS of the guest. The observed acceleration corresponds to a decrease in the bowl-to-bowl inversion barrier of 11.6 kJ mol^−1^ compared to the uncatalyzed process. Comparative binding studies for corannulene (20 π-electrons) and other planar polycyclic aromatic hydrocarbons (PAHs) with 14 to 24 π-electrons were applied to rationalize this barrier reduction. They revealed high binding constants that reach, in tetrachloromethane as a solvent, the picomolar range for the largest guest coronene. Computational models corroborate these experimental results and suggest that both TS stabilization and ground state destabilization contribute to the observed catalytic effect. Hereby, we find a “mutual induced fit” between host and guest in the TS complex, such that mutual geometric adaptation of the energetically favored planar TS and curved π-systems of the host results in an unprecedented non-planar TS of corannulene. Concomitant partial planarization of the PBI units optimizes noncovalent TS stabilization by π–π stacking interactions. This observation of a “mutual induced fit” in the TS of a host–guest complex was further validated experimentally by single crystal X-ray analysis of a host–guest complex with coronene as a qualitative transition state analogue.

## Abstract 2

Combined experimental and computational studies find a perylene bisimide cyclophane to catalyse the corannulene bowl-inversion. Host and guest undergo mutual geometric adaptation, maximizing transition structure stabilization from π–π stacking.

## Graphical abstract

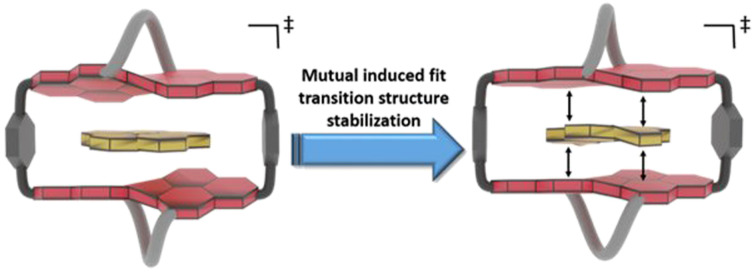

## 1. Introduction

Corannulene,^1–3^ also called [5]circulene, has been known for over 50 years and is the most prominent example of a positively curved PAH.^4,5^ Its bowl shape originates from the strain associated with a five-membered ring surrounded by five six-membered rings. At room temperature, corannulene undergoes fast degenerate bowl-to-bowl inversion^6,7^ with a barrier of approximately 42–46 kJ mol^−1^.^8^ Siegel and co-workers have shown that this inversion barrier of corannulene derivatives is dependent on the bowl depth,^9^ which can be tuned by suitable substitution, *i.e.* benzannulation,^10^ with shallower bowl depths leading to lower inversion barriers. It is well known that this inversion proceeds *via* a fully planar transition structure (TS). Nevertheless, great efforts have been made to obtain flat corannulene as a thermodynamically stable molecule. To this end, it was shown that suitable coordination to a ruthenium complex results in corannulene with a planar geometry.^11^ Furthermore, it was recently demonstrated both computationally and experimentally that sufficiently strong, metal-free stereoelectronic effects, achieved by a suitable substitution, result in an isolable flat corannulene, which is a local minimum (not a TS) on the potential energy surface.^12^

Aside from the synthetic fine-tuning of the bowl depth, some groups have investigated the impact of supramolecular host–guest encapsulation on corannulene's inversion process. In this context, Nitschke and co-workers recently showed how a curved host and second guest cooperatively inhibit the dynamic motion of complexed corannulene.^13^ In contrast, Siegel and co-workers showed that supramolecular complexation in a suitable box-shaped cyclophane could be used to stabilize the planar geometry of corannulene's inversion TS, which accelerates the rate of bowl-inversion,^14,15^*i.e.* supramolecular catalysis.^16,17^ Thus, in 2014 the authors used the corannulene inversion catalyzed by ExBox^4+^, a tetracationic cyclophane consisting of 1,4-phenylene-extended bipyridinium moieties bridged end-to-end by *para*-xylylene units, to provide a conceptually simple supramolecular example of induced fit catalysis: catalysis originates from the reorganization of the host from a strained conformation in the reactant complex to an energetically favorable geometry in the complex with the complementary planar transition structure of the guest.^14^ It has further been shown computationally that adsorption onto 2D materials such as graphene may catalyze the bowl-inversion in a similar fashion.^18–20^ These results show that the kinetics of the inversion process can be controlled both by synthetic modifications and by supramolecular complexation, and that the geometry of corannulene in the ground state can be adjusted by suitable metal coordination or substitution. However, there is still no example of a non-planar transition structure of corannulene's bowl-to-bowl inversion.

**Fig. 1:**
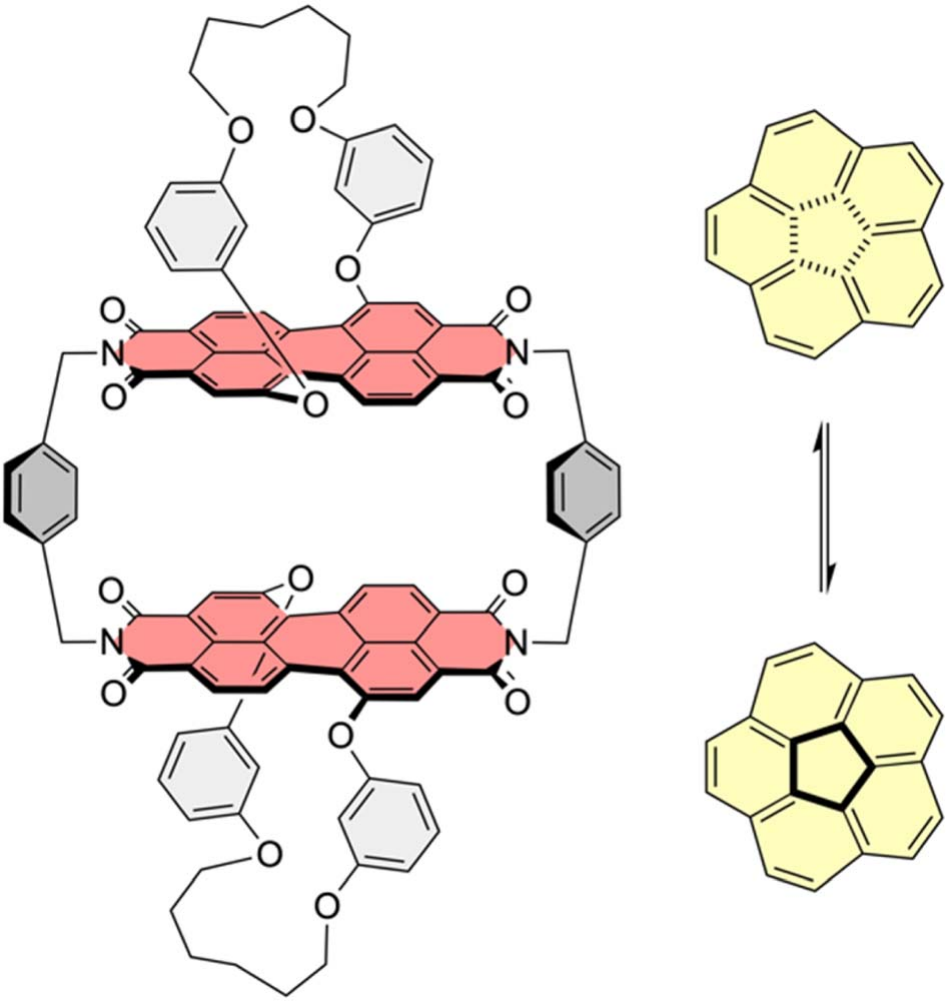
Chemical structure of host 1-*PP* and equilibrium of bowl-to-bowl inversion of corannulene.

We recently reported the synthesis of a PBI cyclophane composed of chiral chromophores,^21^ whose chemical structure is depicted in Fig. 1. This macrocycle proved to be an excellent host molecule for the encapsulation of small carbohelicenes due to an optimal shape complementarity between the helically twisted PBI units and the guests. In-depth studies showed that this host is able to lower the enantiomerization barrier of [5]helicene by acting as a template catalyst.^21,22^ This example of an intricate process proceeding *via* a non-planar TS has drawn our attention to the possible influence of this cyclophane template, composed of rather rigid non-planar PBIs, on the planar TS of corannulene's bowl-to-bowl inversion. Based on the seminal work of Juríček *et al.* on induced fit catalysis,^14^ the frequently discussed concepts of anion-π^23,24^ and cation-π^25^ catalysis, as well as on theoretical work on π–π catalysis^26^ exerted by PBI cyclophanes with planar chromophores on the stereoinversions of polycyclic aromatic hydrocarbons (PAHs) with planar TSs,^27,28^ we were interested in the impact of this host's comparably rigid, non-planar and therefore non-complementary geometry on the dynamic bowl-to-bowl inversion of corannulene.

## 2. Results and discussion

### 2.1 Thermodynamic properties of host–guest complexes

**Fig. 2:**
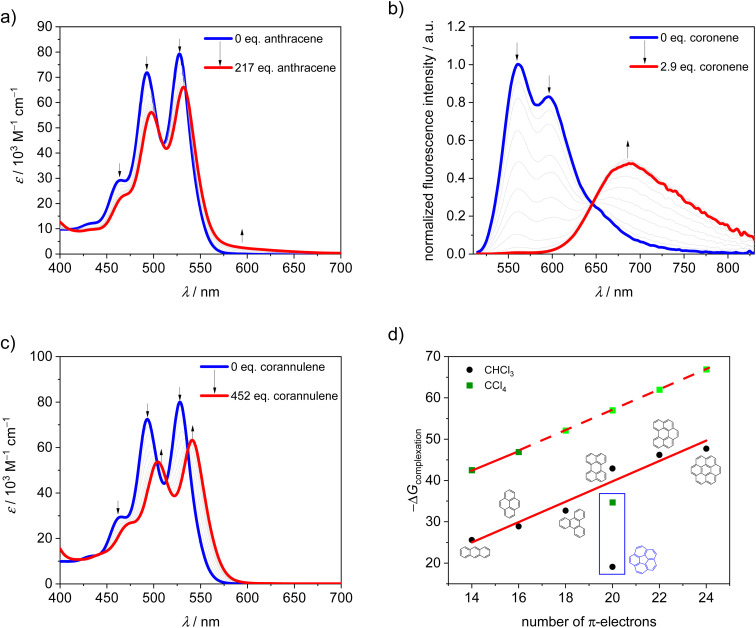
UV/vis titration curves of 1-*PP* upon the addition of (a) anthracene, (b) coronene in chloroform at 295 K and fluorescence titration curve (*λ*_exc_ = 507 nm) of 1-*PP* upon the addition of (c) corannulene in chloroform at 295 K. (d) Plot of Gibbs free binding energy of complexation for various guests by 1-*PP* in chloroform and tetrachloromethane at 295 K. The light green symbols indicate that the values are estimated based on linear extrapolation.

It is well known that in supramolecular host–guest systems where binding site and guest mainly interact *via* π–π stacking, an increase in the number of π-electrons in the guest is correlated with an increase in binding energy.^29–33^ Hence, in order to characterize 1-*PP* as a host for planar substrate recognition, we selected a series of PAH guests with increasing numbers of π-electrons and carried out UV/vis and fluorescence titration studies in chloroform (Fig. S1–S7†). For the smaller guests with lower binding constants, UV/vis spectroscopy was applied to obtain the association constants in chloroform at 295 K. In the corresponding plots of absorption, a decreasing maximum extinction coefficient, well defined isosbestic points as well as an increasing *A*_0–0_/*A*_0–1_ ratio of the PBI S_0_–S_1_ transition can be observed (Fig. 2a and S1–S3†). Hence, guest encapsulation interrupts the H-type excitonic coupling between the two PBI chromophores by increasing the distance between them.^34,35^ Accordingly, the shape of the spectra of the complexes is more reminiscent of a monomeric PBI.^36^ Furthermore, a pronounced red-shifted shoulder is apparent for some of the host–guest complex spectra which corresponds to a charge transfer between the electron poor PBIs and the electron rich guests like anthracene and pyrene.^31^ From the data evaluation, *i.e.* non-linear curve fitting of the changes in absorption, an increase in the binding constant from 3.4 × 10^4^ M^−1^ for anthracene (14 π-electrons) to 6.1 × 10^5^ M^−1^ for triphenylene (18 π-electrons) could be determined.

For the larger guests, *i.e.* perylene, benzo[*ghi*]perylene and coronene, fluorescence titration studies were performed. While only a fluorescence turn-off is revealed upon the addition of perylene (20 π-electrons, Fig. S4†), an exciplex band emerges for benzo[*ghi*]perylene complexation (22 π-electrons, Fig. S5†). This band is further increased in intensity for coronene (24 π-electrons, Fig. 2b and S6†) as the largest guest, supporting a significant charge transfer character for these complexes. The corresponding complexation energies fit the expected linear increase with a larger size of the guests' aromatic surfaces (Fig. 2d). For coronene, the binding constant in chloroform reaches a remarkably high value of *K*_a_ = 2.8 × 10^8^ M^−1^. Upon decreasing the solvent polarity, the binding constants can be further increased for this host if chloroform is replaced by tetrachloromethane as a solvent. For anthracene and pyrene, the association constants could be increased by three orders of magnitude (Fig. S8 and S9†). If a linear free energy relationship is assumed for all PAH guests for the transition from chloroform to tetrachloromethane, the Gibbs free energy can be extrapolated for the larger guests (Fig. 2d). This yields a complexation Gibbs free energy of 66.9 kJ mol^−1^ for coronene in tetrachloromethane, corresponding to an impressive binding constant of around 10^12^ M^−1^ in this organic solvent. Table 1 summarizes the binding constants for the guest complexations by 1-*PP* and the corresponding Gibbs free energies in chloroform and tetrachloromethane.

**Table 1:** Binding constants for the encapsulation of several guests by 1-*PP* determined from global nonlinear curve fit routine of UV/vis or fluorescence data at 295 K, as well as the corresponding Gibbs free energies of complexation

Guest	*K*_a_ (CHCl_3_) [M^−1^]	−Δ*G*^0^ (CHCl_3_) [kJ mol^−1^]	*K*_a_ (CCl_4_) [M^−1^]	−Δ*G*^0^ (CCl_4_) [kJ mol^−1^]
Anthracene	3.4 × 10^4^	25.6	3.3 × 10^7^	42.5
Pyrene	1.3 × 10^5^	28.9	2.0 × 10^8^	46.9
Triphenylene	6.1 × 10^5^	32.7	1.7 × 10^9^^a^	52.1^a^
Perylene	4.0 × 10^7^	42.9	1.2 × 10^10^^a^	57.0^a^
Benzo[*ghi*]perylene	1.5 × 10^8^	46.2	9.5 × 10^10^^a^	62.0^a^
Coronene	2.8 × 10^8^	47.7	7.0 × 10^11^^a^	66.9^a^
Corannulene	2.4 × 10^3^	19.1	1.4 × 10^6^	34.7

a Numbers are estimated based on linear extrapolation of the Gibbs free energy in Fig. 2d and calculation of the binding constants according to 
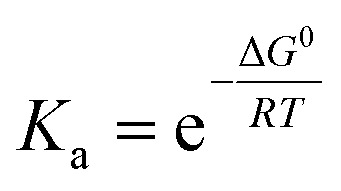
.

Thus, despite the apparent structural mismatch between the helically chiral cavity of the host and the planar guests, we find extraordinarily high binding affinities for the complexes with planar PAHs. In contrast, curved corannulene (20 π-electrons) shows a much weaker binding by 1*-PP*. The optical changes in the corresponding UV/vis titration study (Fig. 2c and S7†) are qualitatively comparable to the one of triphenylene (Fig. S3†), which has 18 π-electrons. However, with a binding constant of *K*_a_ = 2.4 × 10^3^ M^−1^ in chloroform at 295 K, the complexation energy is by 13.6 kJ mol^−1^ lower, despite the larger aromatic surface of corannulene. Indeed, based on the linear relationships shown in Fig. 2 we can calculate Gibbs free energies for a planar 20 π-electron PAH guest of 42.9 kJ mol^−1^ in chloroform and 57.0 kJ mol^−1^ in tetrachloromethane that are significantly larger than those observed for the bowl-shaped corannulene (Table 1).

### 2.2 Structural properties of host–guest complexes

**Fig. 3:**
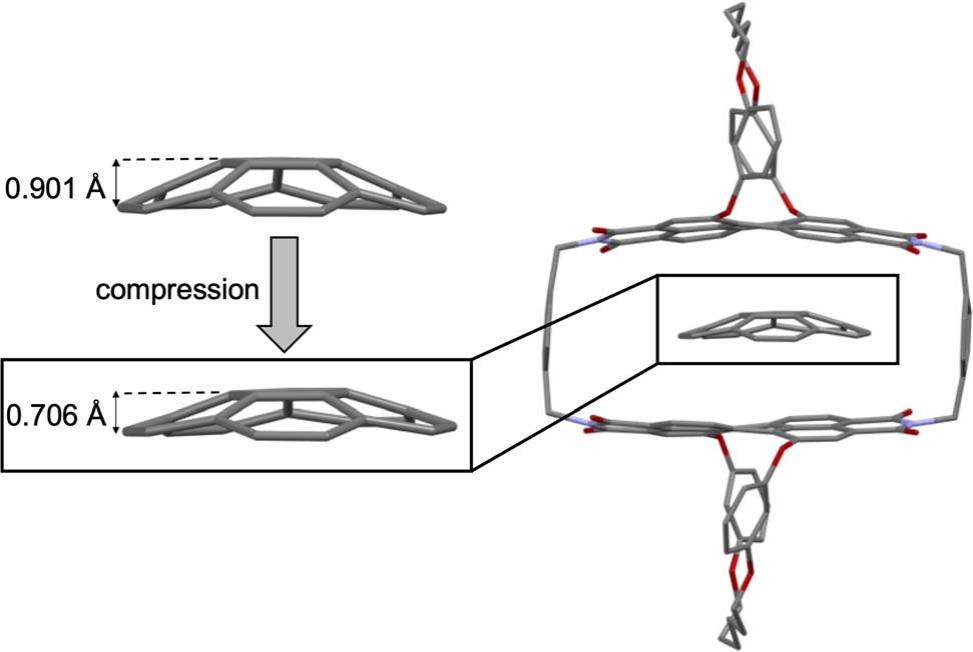
Structures of free corannulene and corannulene⊂1-*PP* optimized at the B3LYP-D3(BJ)/def2-SVP level of theory along with a zoom-in on the guest to illustrate its compression upon complexation.

Computational studies of corannulene⊂1-*PP* result in a binding energy for this complex of 41.1 kJ mol^−1^ in tetrachloromethane at the SMD-(CCl_4_)-PW6B95-D3(BJ)/def2-TZVPP level of theory,^37–45^ which is in reasonably good agreement with the experimental value (*vide supra*). The optimized geometry of the corannulene⊂1-*PP* complex reveals that while the chiral PBI host accommodates the guest well, corannulene is significantly compressed by the chromophore planes, resulting in a significant decrease in bowl depth by as much as 0.195 Å compared to the calculated structure of free corannulene (Fig. 3). Furthermore, a comparison of the core twists of the PBIs shows that complexation with corannulene either results in an almost unchanged twist (18.5°, PBI facing the convex side of corannulene) or induces a decreased twist (12.3°, PBI facing the concave side of corannulene) compared to the free host (average of 18.1°, see Table S2† for details), illustrating the structural adaptivity of both, the cyclophane host and the bowl-shaped guest.

**Fig. 4:**
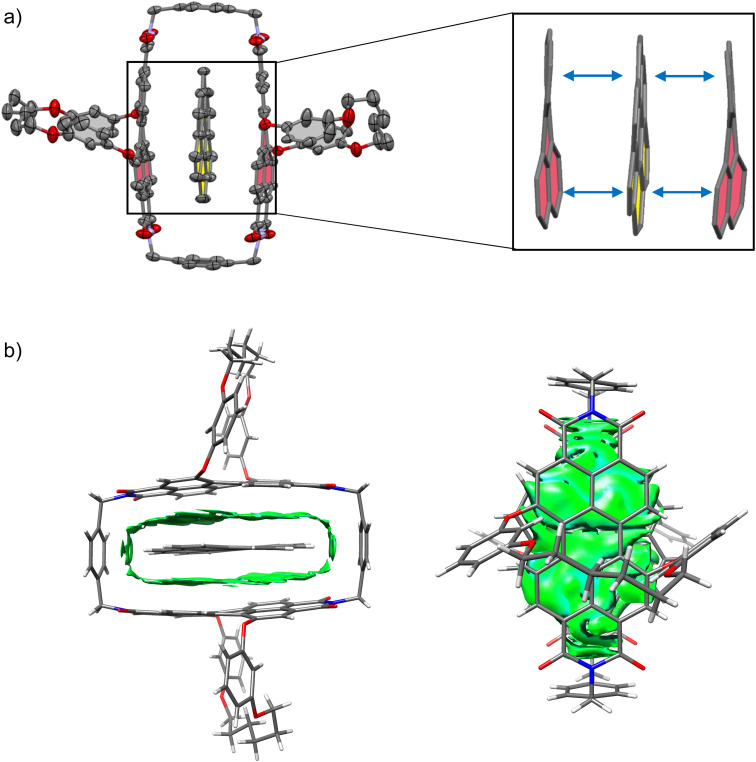
(a) Molecular structure of coronene⊂1-*PP* complex, obtained from single crystal X-ray analysis (thermal ellipsoids set at 50% probability) with enlarged excerpt, showing how the perylene units and the guest maximize their interaction by a mutual geometric adaption. The guest is highlighted in yellow, the perylene units are highlighted in red. Solvent molecules and hydrogen atoms are omitted for clarity. (b) Noncovalent interactions (NCI) plot of the single crystal structure of coronene⊂1-*PP*.

The studies on the guest binding by 1-*PP* revealed that high binding energies can be achieved for the encapsulation of flat PAHs despite the twisted nature of the PBI units in the cyclophane. To obtain further insights into the structures of these complexes, we grew a co-crystal from a mixture of coronene, the most strongly bound guest, and 1-*PP* by slow evaporation of *n*-hexane into a chlorobenzene solution. The resulting crystal could be analyzed by single crystal X-ray analysis (Fig. 4a). Crystallographic analysis showed that the complex crystallizes in the triclinic system (for details see the ESI†). The crystal structure not only clearly confirms the intercalation of coronene between the twisted PBIs, but also reveals a distorted geometry of flat coronene within the complex. Furthermore, the twist of the PBI's naphthalene subunits shows a flattening of the perylene core with a small twist angle of 12.8° (average from both chromophores of the two complexes in the unit cell). Hence, the loss of planarity of coronene is accompanied by a reduction in the PBI core twists. The intermolecular contacts between the twisted perylene units and the distorted coronene guest were further qualitatively investigated with the help of NCI (noncovalent interactions) analysis^46^ of the co-crystal (Fig. 4b).

The green regions in the NCI plot can be attributed to van der Waals interactions between the host and the guest and illustrate how the induced structural adaption in this complex optimizes noncovalent interactions between the aromatic planes of the guest and the perylene bisimides. The additional stabilizing CH–π interactions between coronene and the *para*-xylylene spacer units further become apparent. When inspecting the NCI plot from a top-down perspective (Fig. 4b, right), the intermolecular binding iso-surface also reveals that almost the full aromatic surface of coronene contributes to the host–guest binding. This explains the continuous increase of the complexation Gibbs free energy with substrate size even beyond perylene, which could otherwise be presumed as the perfect guest for this host.

### 2.3 Catalysis of bowl-to-bowl inversion

Despite the structural mismatch between flat guests and core-twisted PBIs, we considered this host to be suitable for catalyzing corannulene's bowl-to-bowl inversion. According to our binding studies (Fig. 2d and Table 1) we could indeed expect a substantial transition structure stabilization from the differences of the calculated Gibbs free energies for a planar 20 π-electron guest and the experimental value for corannulene. Further, the single crystal X-ray analysis revealed that coronene, which is a qualitative transition state analogue for the bowl-to-bowl inversion of corannulene, is complexed with a mutual induced structural adaption of host and guest. Thus, the planar TS of corannulene should experience more stabilization within the cyclophane cavity than the curved equilibrium structure, which should result in catalysis of the stereoinversion.

To study the dynamic bowl-to-bowl inversion process, the alkyl substituent of ethylcorannulene proved a good diagnostic group in order to quantify the inversion of corannulene as the methylene protons become heterotopic at low temperatures, when the bowl-to-bowl motion is slow on the NMR time scale.^14^ Accordingly, variable temperature NMR studies were performed in deuterated dichloromethane (DCM-*d*_2_), which due to its low melting point is more suitable than chloroform or tetrachloromethane to cover the whole dynamic process. We note that the additional ethyl substituent had no impact on the binding affinity, which was tested by host–guest titration and subsequent data evaluation (Fig. S11 and S12†). We further note in this context that the UV/vis absorption spectrum of the complex in tetrachloromethane is in good agreement with the one in DCM (Fig. S14†), which indicates that no structural changes in the host–guest assembly are induced by the less polar solvent but only weak solvatochromism is observed.

The binding affinity for corannulene is weaker in DCM than in chloroform. This was also confirmed for the most strongly bound guest coronene (Fig. S13†), indicating that this is likely to be a general solvent effect. As only one set of signals results for the host protons even at temperatures below 170 K for a host-ethylcorannulene mixture with 2 eq. of 1-*PP* (Fig. S19†), guest exchange is fast compared to the bowl-to-bowl inversion over the whole temperature range needed for the studies on this process. Hence, the bowl-to-bowl motion is the rate-determining step which allows direct conclusions on the guest inversion from the dynamic NMR data not only in the absence but also in the presence of the host template and we expect the solvent effect to not affect the studies on the kinetics of interest.

**Fig. 5:**
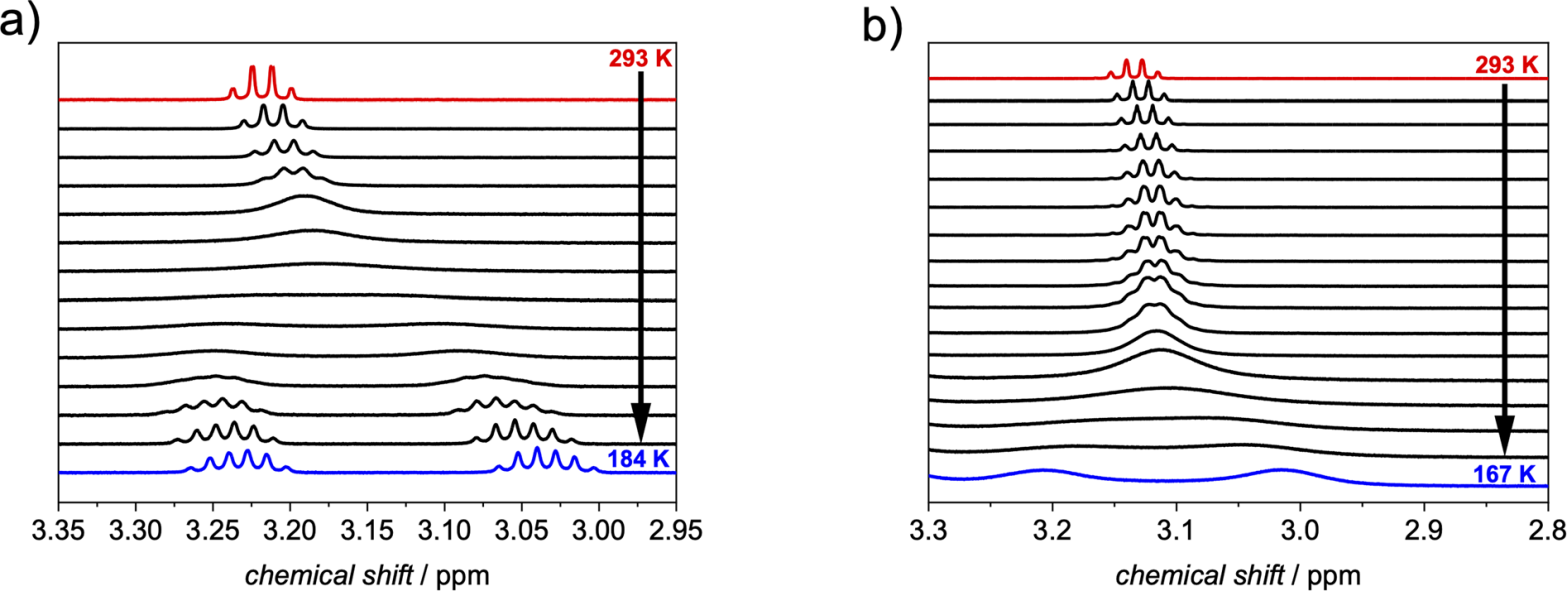
Excerpt from variable temperature ^1^H NMR of ethylcorannulene in the (a) absence and in the (b) presence of 0.5 equivalents of 1-*PP* (*c* (1-*PP*) ≈ 1 mM, exact temperatures are given in the ESI†).

At room temperature, a quartet for the methylene protons of the free substrate is apparent, which becomes more and more broadened upon cooling down until the coalescence at 233 K is reached. At lower temperatures these protons split into two signals. For free ethylcorannulene (Fig. 5a and S15†), an inversion barrier of 45.8 kJ mol^−1^ could be derived from the coalescence temperature and the signal splitting according to eqn (S1†), which is in very good agreement with the literature value for corannulene.^8^ A similar temperature series was carried out in the presence of 0.5 eq. of 1-*PP* (Fig. 5b and S16†), revealing this barrier to be decreased by around 25% to 34.2 kJ mol^−1^ with a coalescence temperature of 176 K for the methylene protons of the guest. This decrease of the barrier towards inversion was further supported by VT experiments with more (Fig. S17†) or less (Fig. S18†) than 0.5 equivalents of the host, which had only minor impact on the catalytic effect: while two equivalents of 1-*PP* lowered the barrier to 33.7 kJ mol^−1^, supporting the fact that the guest exchange is fast compared to the bowl-to-bowl inversion (*vide supra*), we determined a slightly higher barrier of 36.8 kJ mol^−1^ with 0.1 equivalents of host. These changes might be attributable to the slow diffusion of the reactants at very low temperatures close to the freezing point of dichloromethane, when the solvent gets more viscous.^47^

**Fig. 6:**
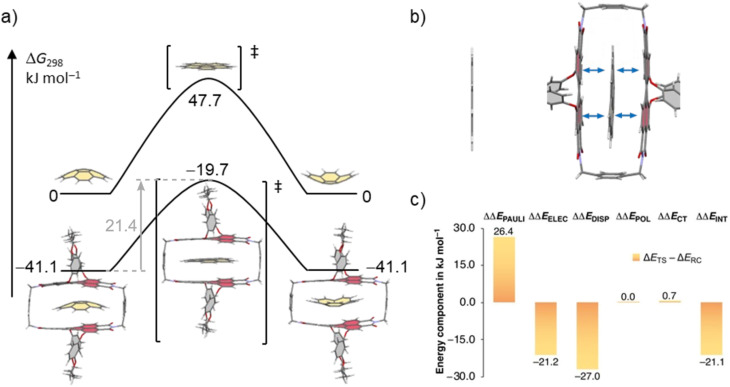
(a) Schematic potential energy surface (PES) of the uncatalyzed and the catalyzed bowl-to-bowl inversion of corannulene in the absence and the presence of 1-*PP* in CCl_4_ with Gibbs free energies given relative to the free host and guest. Geometries and energies were obtained at the SMD-(CCl_4_)-PW6B95-D3(BJ)/def2-TZVPP//B3LYP-D3(BJ)/def2-SVP level of theory. (b) Calculated transition structure for the bowl-to-bowl inversion of corannulene and corannulene⊂1-*PP*. The 1,7-bridging motif in the cyclophane is cut off for clarity. (c) Breakdown of relative noncovalent stabilizations and destabilizations of TSs (ΔΔ*E* = Δ*E*_TS_ − Δ*E*_RC_) from ALMO-EDA-II analysis^48^ at the ωB97M-V/def2-SVP level of theory into contributions from Pauli repulsions (ΔΔ*E*_PAULI_), electrostatic interactions (ΔΔ*E*_ELEC_), dispersion interactions (ΔΔ*E*_DISP_), polarization (ΔΔ*E*_POL_), and charge transfer (ΔΔ*E*_CT_).

In order to rationalize the observed acceleration of the bowl-to-bowl inversion within the cyclophane host, we performed computational studies of the uncatalyzed and host–guest catalyzed processes at the SMD-(CCl_4_)-PW6B95-D3(BJ)/def2-TZVPP//B3LYP-D3(BJ)/def2-SVP level of theory.^37–45^Fig. 6 shows the resulting schematic potential energy profiles for the two processes, as well as a breakdown of the contributions to relative noncovalent stabilizations and destabilizations of transition structures obtained from second generation absolutely localized molecular orbital (ALMO) energy decomposition analyses (EDA).^48^

For the bowl-inversion of free corannulene, we obtain a Gibbs free energy barrier of 47.7 kJ mol^−1^, which is in good agreement with the experimentally obtained value of 45.8 kJ mol^−1^ (Fig. 6a). Further, in qualitative agreement with the experimental data we find complexation of corannulene with 1-*PP* to lead to a notable reduction in the bowl-to-bowl inversion barrier. We note that in comparison to the experimentally determined bowl-inversion barrier upon complexation of 34.2 kJ mol^−1^, we computationally obtain a lower barrier of 21.4 kJ mol^−1^, indicating that computationally we predict a larger catalytic effect. This discrepancy between theory and experiment is attributed to the inaccuracy of the DFT methods and implicit solvent model employed and to aspects of the experimental system partially captured by the computational simulations. As such, a recent study estimating the catalytic effect of ExCage^6+^ on the bowl-inversion of indenocorannulene reported that due to issues of low substrate concentration and low binding affinity, the observed rate constant of enantiomerization was by approximately two orders of magnitude smaller than the real rate constant of the inversion occurring within the host, *i.e.* the process that can be modelled computationally.^15^ Due to temperature requirements of the variable temperature NMR studies, we here use DCM, a competitive solvent expected to give lower binding affinities, close to its freezing point when determining the inversion barrier experimentally. It is possible that this may have a similar effect on the observed rate and resulting experimental barrier as reported in ref. 15. Most notably, however, we find the TS in the optimized complex with 1-*PP* to show the same unusual distortion from planarity that we observed experimentally for the complex between 1-*PP* and the TS analogue coronene (Fig. 4). We equally find that this geometric change in the guest goes along with a flattening of the PBI moieties of the host (Fig. 6b and Table S2†). Energy decomposition analyses (Fig. 6c and Table S3†) show that compared to the reactant complex, noncovalent interactions between the guest and the perylene planes are indeed maximized in the TS complex, in which host and guest geometries mutually adapt to optimize π–π stacking interactions. Such “mutual induced fit transition structure stabilization” is a well conceivable explanation for the observed catalytic effect of this host for the corannulene bowl-inversion, despite the initial non-complementarity between the non-planar PBI chromophores and the planar guest TS. In order to gain quantitative insights into the energetic consequences of this geometric adaptation upon complexation, we obtain an estimate of the contribution of ground state destabilization to the catalytic effect based on our optimized geometries. Table 2 provides the differences in electronic energies between the geometries of the free optimized corannulene equilibrium and transition structures and PBI cyclophane, and the corresponding geometries of the catalyst and substrate resulting from optimizations of the host–guest complexes. The difference between the sums of the total destabilizations to host and guest in the ground and transition state complexes provides an estimate of the contribution of ground state destabilization to catalysis.

**Table 2:** Summary of destabilizations of host and guest for ground state (GS) and transition state (TS) structures given as the differences in electronic energies in kJ mol^−1^ between the free equilibrium structures of corannulene and 1-*PP* and the corresponding geometries of the isolated host and guest structures in their geometries in the reactant and TS complexes

GS_host_	GS_guest_	TS_host_	TS_guest_	∑GS	∑TS	GS-TS^a^
2.5	7.7	2.0	0.5	10.2	2.5	7.7

a Corresponds to the effective ground state destabilization that contributes to catalysis.

As apparent from Table 2, our calculations indicate that apart from transition structure stabilization, ground state destabilization contributes to the observed catalytic effect. While overall distortions upon complexation only result in a total destabilization of 2.5 kJ mol^−1^ in the TS complex, we obtain a total destabilization of 10.2 kJ mol^−1^ for the reactant complex. Thus, we arrive at a contribution of ground state destabilization to catalysis of 7.7 kJ mol^−1^. It is instructive to note that this contribution is larger than the contribution of ground state destabilization of 2.1 kJ mol^−1^ reported for the ExBox^4+^ catalyzed corannulene bowl-inversion.^14^ This is presumably a consequence of the large, rigid PBI π-surface of this host compared to the smaller, more flexible 1,4-phenylene-extended bipyridinium moieties of ExBox^4+^. Hereby, the energy difference between the freely optimized corannulene bowl and its geometry in the complex with 1-*PP* is, at 7.7 kJ mol^−1^, the main contribution to destabilization. Inspection of the corresponding structures reveals that this is mainly the result of a reduction in bowl-depth upon complexation, with the distance between the centroids of the central 5-membered ring to the centroid of the 10 atoms forming the corannulene rim decreasing from 0.901 Å to 0.706 Å (Fig. S21†). By comparison, our calculations reveal that despite the unusual deviation from planarity of the bowl-inversion TS in the complex, this geometric adaptation carries almost negligible energetic penalty (0.5 kJ mol^−1^), explaining why catalysis is observed despite the need for mutual induced fit binding in the transition structure complex.

## 3. Conclusion

In summary, we have reported strong binding of planar PAHs and π–π catalysis of corannulene's bowl-to-bowl inversion within a cyclophane cavity consisting of twisted PBI chromophores. Computational models of the catalytic process reveal a non-planar geometry of corannulene in the TS when complexed by 1-*PP*, which in turn partially planarizes. Thus, in the case of [corannulene⊂1-*PP*]^‡^ not only the core twists of the PBI chromophores in the macrocycle adapt to the favored planar transition structure of the guest but the guest also loses its planar geometry, maximizing the stabilizing π–π stacking interactions between the π-systems of host and guest. This observation was supported experimentally by single crystal X-ray analysis of a coronene⊂1-*PP* complex as a transition state analogue and significantly stronger binding of planar PAHs by 1-*PP*, *i.e.* approximately five orders of magnitude stronger binding of coronene in chloroform compared to corannulene. In less polar tetrachloromethane, the binding constant for coronene reaches the exceptionally high value of around 10^12^ M^−1^. Accordingly, the solution studies confirm the preferred binding of more planar aromatic guests compared to curved corannulene while insights from the solid-state structure indicate a mutual adaption of guest and host and thereby a mutual induced fit transition structure stabilization for the bowl-to-bowl inversion of corannulene.

## Data availability

The experimental procedures, analytical data, and computational details are available within the manuscript and its ESI file.† CCDC number 2300009 contains the supplementary crystallographic data for the coronene⊂1-*PP* complex. Additional data underlying this study are openly available in Zenodo, see https://doi.org/10.5281/zenodo.10184971.

## Author contributions

MW, AAK, AK, and FW designed the research, MW performed syntheses and carried out experimental characterizations and analyses, OA performed crystallographic analyses, AAK performed DFT calculations, MW and AAK wrote the manuscript with review and editing by AK and FW.

## Conflicts of interest

There are no conflicts to declare.

## Supplementary Material

SC-015-D3SC05341E-s001

SC-015-D3SC05341E-s002
