# Supplementary material for: Mutual induced fit transition structure stabilization of corannulene's bowl-to-bowl inversion in a perylene bisimide cyclophane
Source: Chem Sci. 2023 Nov 27;15(2):609–17. doi: 10.1039/d3sc05341e (PMC10762775; doi:10.1039/d3sc05341e)
Supplement: SC-015-D3SC05341E-s001 [file SC-015-D3SC05341E-s001.pdf]

Electronic Supplementary Material (ESI) for Chemical Science.
This journal is © The Royal Society of Chemistry 2023

Mutual Induced Fit Transition Structure Stabilization of Corannulene's Bowl-to-Bowl Inversion in a Perylene Bisimide Cyclophane

Supplementary Information

Manuel Weh,[†] Asja A. Kroeger,[§] Olga Anhalt,[‡] Amir Karton,[*, §, ⹋] and Frank Würthner[*, †, ‡]

[†]Institut für Organische Chemie, Universität Würzburg, Am Hubland, 97074 Würzburg, Germany

[§]School of Molecular Sciences, The University of Western Australia, 35 Stirling Highway, Crawley, WA 6009, Australia

[‡]Center for Nanosystems Chemistry & Bavarian Polymer Institute, Universität Würzburg, Theodor-Boveri-Weg, 97074 Würzburg, Germany

[⹋]School of Science and Technology, University of New England, Armidale, NSW 2351, Australia

M. W. and A. A. K. contributed equally.

*E-mail: wuerthner@uni-wuerzburg.de

amir.karton@une.edu.au

Table of Content

1. General Methods 3
2. Titration Studies in Chloroform 4
3. Titration Studies in Tetrachloromethane 9
4. Titration Studies in Dichloromethane 11
5. Variable Temperature NMR Studies 13
6. Single Crystal X-Ray Analysis 19
7. DFT Calculations 21
8. References 43

1. General Methods

Chemicals: All chemicals and solvents were purchased from commercial suppliers and used without further purification. **1-*PP*** and ethylcorannulene were synthesized according to literature known procedures.[1-3]

NMR spectroscopy: ^{1}H NMR spectra were recorded on a Bruker Avance III HD 600 MHz spectrometer. Chemical shift data are reported in parts per million (ppm, δ scale) downfield from tetramethylsilane and referenced internally to the residual proton (for proton NMR) in the solvent (CD_2Cl_2: $\delta = 5.32$).

UV/vis absorption spectroscopy: All spectroscopic measurements were carried out under ambient conditions using solvents of spectroscopic grade. The absorption spectra were recorded on a JASCO V-770 or V-670 spectrometer equipped with a PAC-743R Peltier for temperature control.

Steady-State Fluorescence Spectroscopy: Fluorescence spectra were recorded on an Edinburgh Instruments FLS981 fluorescence spectrometer.

Single crystal X-ray analysis: The diffraction images for X-ray crystallographic analysis were collected on a Bruker D8 Quest Kappa diffractometer with a Photon II CMOS detector and multi-layered mirror monochromated Cu Kα radiation. The solvent molecules had to be partially removed by a SQUEEZE routine.[4]

DFT calculations: Geometry optimizations of all equilibrium and transition structures along the PESs of the inversion processes of interest were performed at the B3LYP-D3(BJ)/def2-SVP level of theory.[5-11] Subsequent frequency calculations at the same level of theory were employed to confirm the resulting structures as equilibrium structures (all real frequencies) or transition structures (one imaginary frequency along the reaction coordinate of the bowl-inversion of interest). The connectivities of the optimized transition structures to the respective reactants and products of interest were further confirmed via intrinsic reaction coordinate (IRC) calculations[12, 13] at the B3LYP-D3(BJ)/def2-SVP level of theory. Single point energy calculations using the hybrid meta-GGA functional PW6B95-D3(BJ) in conjunction with the def2-TZVPP basis set were subsequently performed on all optimized structures in order to obtain more accurate supramolecular complexation energies and inversion barriers.[9-11, 14] Corrections for bulk solvent effects of tetrachloromethane were included using the SMD continuum solvation model[15] at the recommended[15] M05-2X/6-31G(d) level of theory. More

detailed insights into noncovalent interaction energies were obtained using the second generation of the Absolutely Localised Molecular Orbital (ALMO) energy decomposition analysis (EDA) scheme[16] at the recommended ωB97M-V/Def2-SVP level. All geometry optimizations, frequency, IRC and single point energy calculations were performed using the Gaussian16 software package.[17]

Titration Studies: For the titration experiments, a solution of PBI cyclophane **1-*PP*** and the respective guest in excess (see corresponding graphs for exact amount of the individual guest) was titrated to a solution of the pure cyclophane in the same solvent (mixture) of the same concentration keeping the host concentration constant during the experiment. The UV/vis or fluorescence titration data were fitted to a 1:1 binding model.[18] Data evaluation was furthermore performed by using *bindfit*.[19]

2. Titration Studies in Chloroform

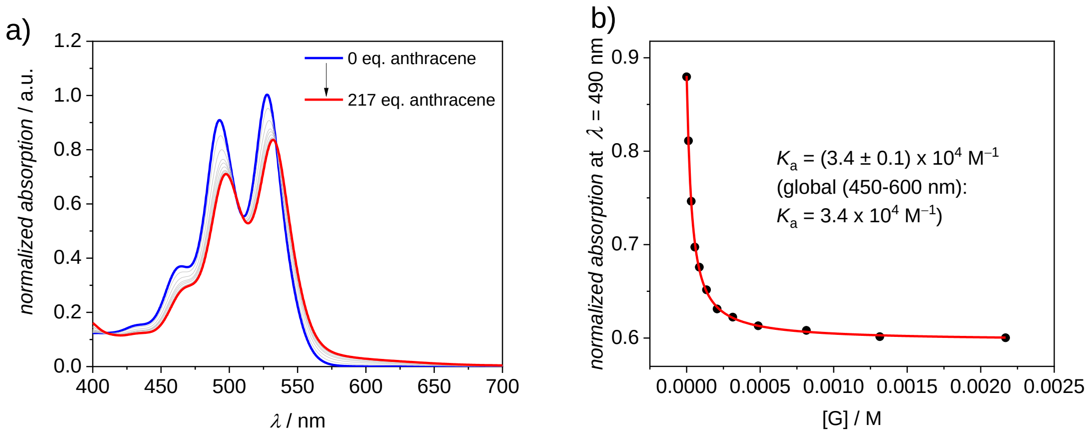

Fig. S1. a) UV/vis spectra of cyclophane **1-*PP*** in $CHCl_3$ at 22 °C (c = 10 x 10^{-6} M) upon the addition of anthracene as a guest and b) the resulting plot of the absorption at λ = 490 nm with nonlinear curve fit (1:1 binding model, red curve).

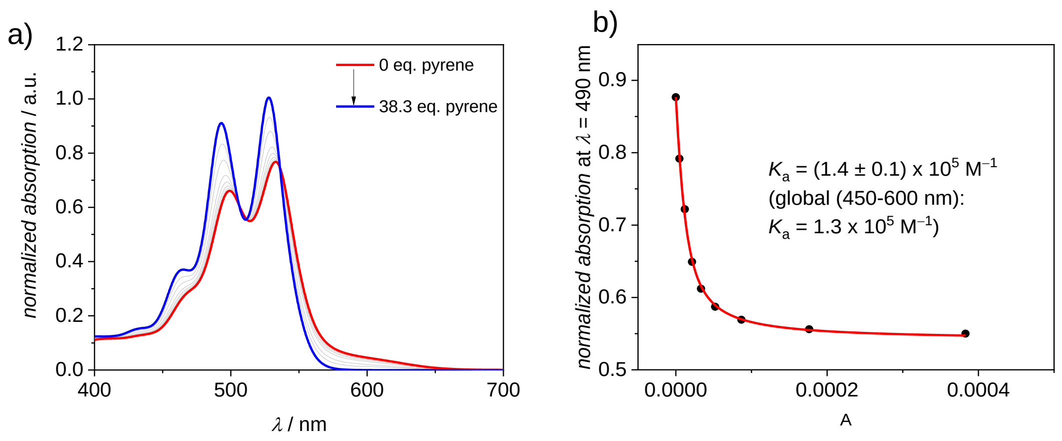

Fig. S2. a) UV/vis spectra of cyclophane **1-*PP*** in $CHCl_3$ at 22 °C (c = 10 x 10^{-6} M) upon the addition of pyrene as a guest and b) the resulting plot of the absorption at λ = 490 nm with nonlinear curve fit (1:1 binding model, red curve).

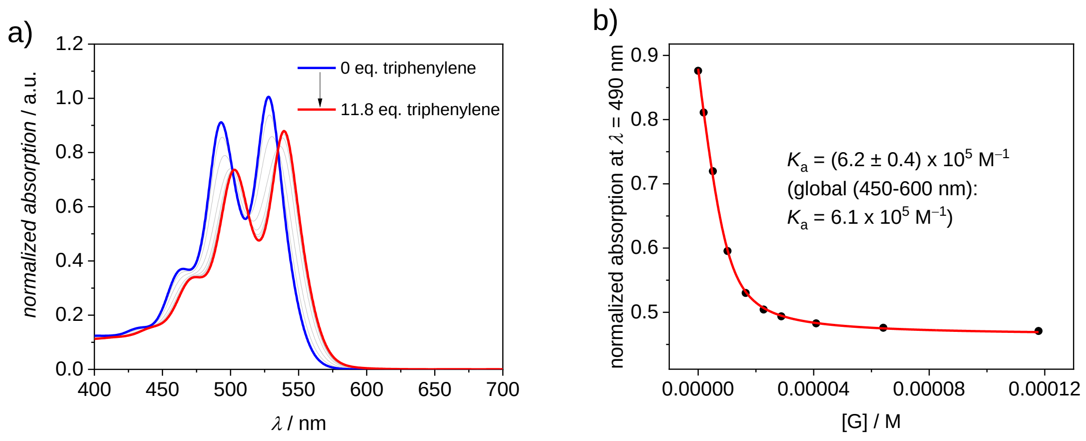

Fig. S3. a) UV/vis spectra of cyclophane **1-*PP*** in $CHCl_3$ at 22 °C (c = 10 x 10^{-6} M) upon the addition of triphenylene as a guest and b) the resulting plot of the absorption at λ = 490 nm with nonlinear curve fit (1:1 binding model, red curve).

a)

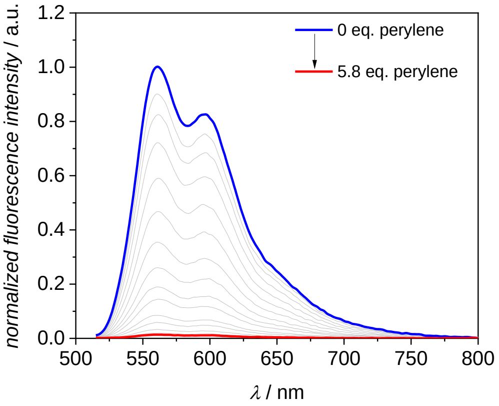

b)

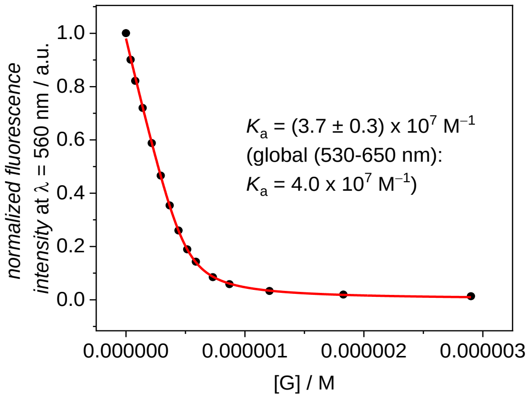

c)

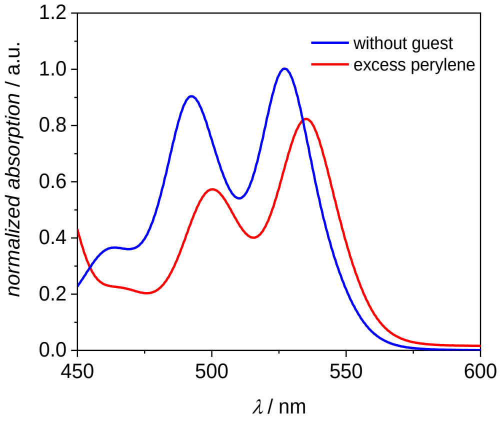

Fig. S4. a) Fluorescence spectra (λ_{exc} = 507 nm) of cyclophane **1-*PP*** in $CHCl_3$ at 22 °C (c = 5 x 10^{-7} M) upon the addition of perylene as a guest and b) the resulting plot of the fluorescence at λ = 560 nm with nonlinear curve fit (1:1 binding model, red curve). c) UV/vis spectrum of free **1-*PP*** and perylene⊂**1-*PP*** in chloroform at 22 °C.

b)

a)

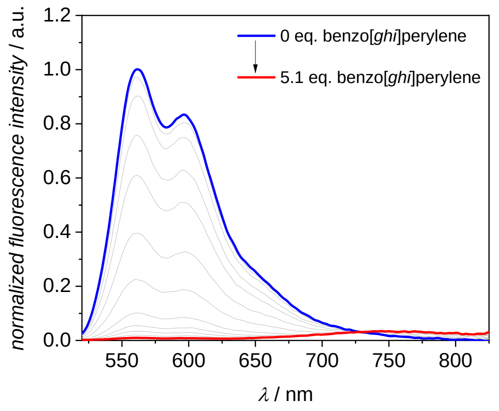

c)

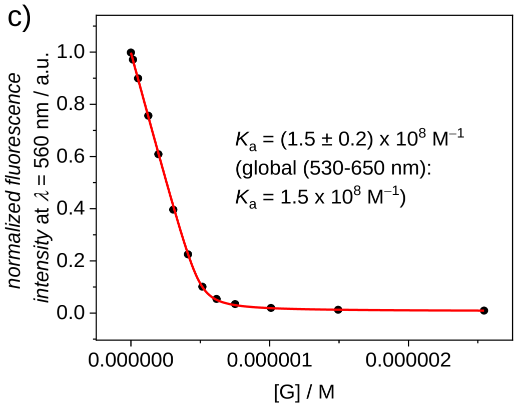

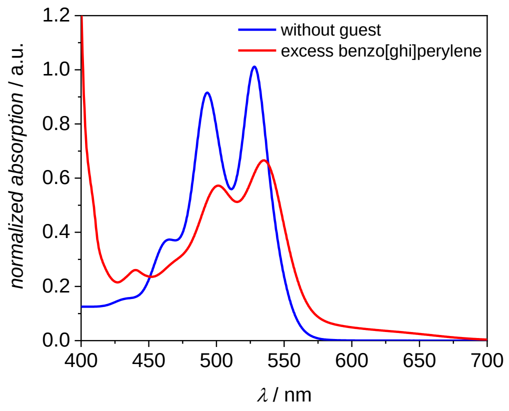

Fig. S5. a) Fluorescence spectra (λ_{exc} = 510 nm) of cyclophane **1-*PP*** in $CHCl_3$ at 22 °C (c = 5 x 10^{-7} M) upon the addition of benzo[*ghi*]perylene as a guest and b) the resulting plot of the fluorescence at λ = 560 nm with nonlinear curve fit (1:1 binding model, red curve). c) UV/vis spectrum of free **1-*PP*** and benzo[*ghi*]perylene⊂**1-*PP*** in chloroform at 22 °C.

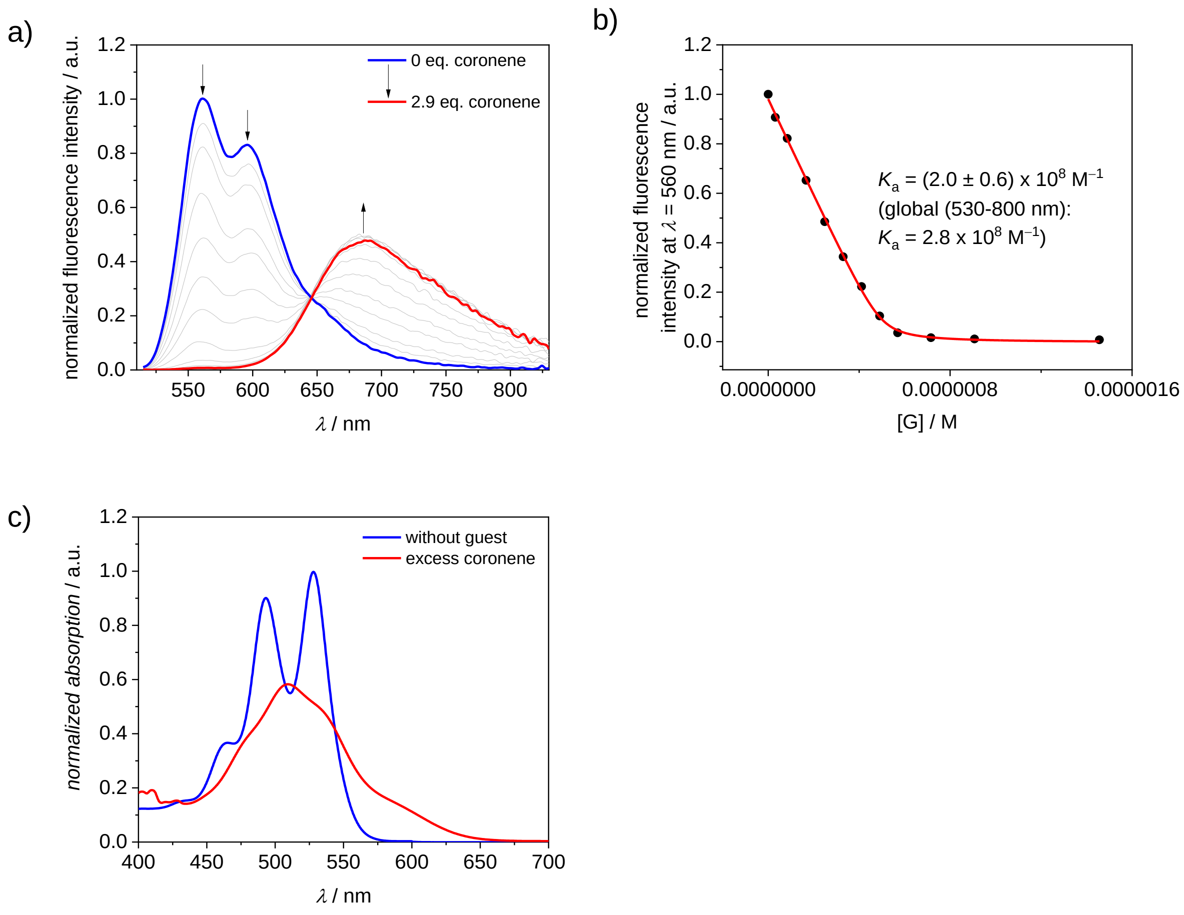

Fig. S6. a) Fluorescence spectra (λ_{exc} = 507 nm) of cyclophane **1-*PP*** in $CHCl_3$ at 22 °C ($c = 5 \times 10^{-7}$ M) upon the addition of coronene as a guest and b) the resulting plot of the fluorescence at λ = 560 nm with nonlinear curve fit (1:1 binding model, red curve). c) UV/vis spectrum of free **1-*PP*** and coronene⊂**1-*PP*** in chloroform at 22 °C.

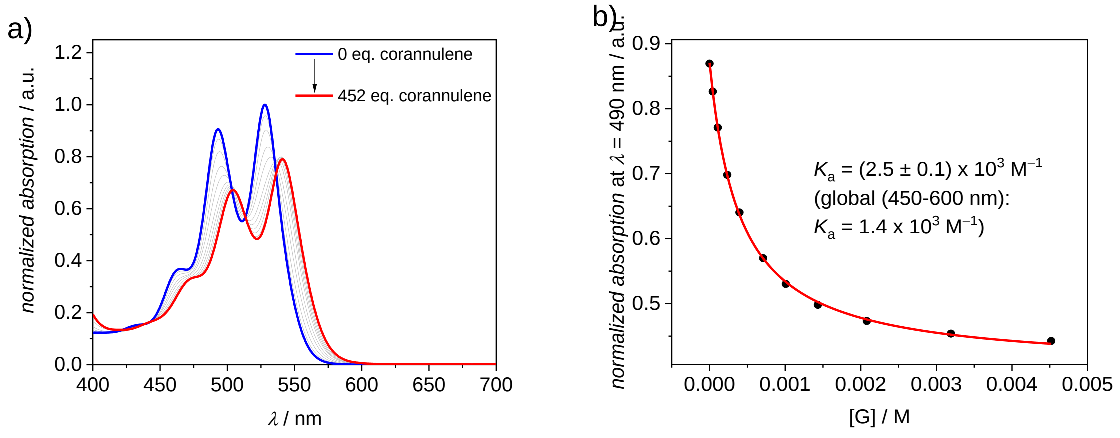

Fig. S7. a) UV/vis spectra of cyclophane **1-*PP*** in $CHCl_3$ at 22 °C ($c = 10 \times 10^{-6}$ M) upon the addition of corannulene as a guest and b) the resulting plot of the absorption at λ = 490 nm with nonlinear curve fit (1:1 binding model, red curve).

3. Titration Studies in Tetrachloromethane

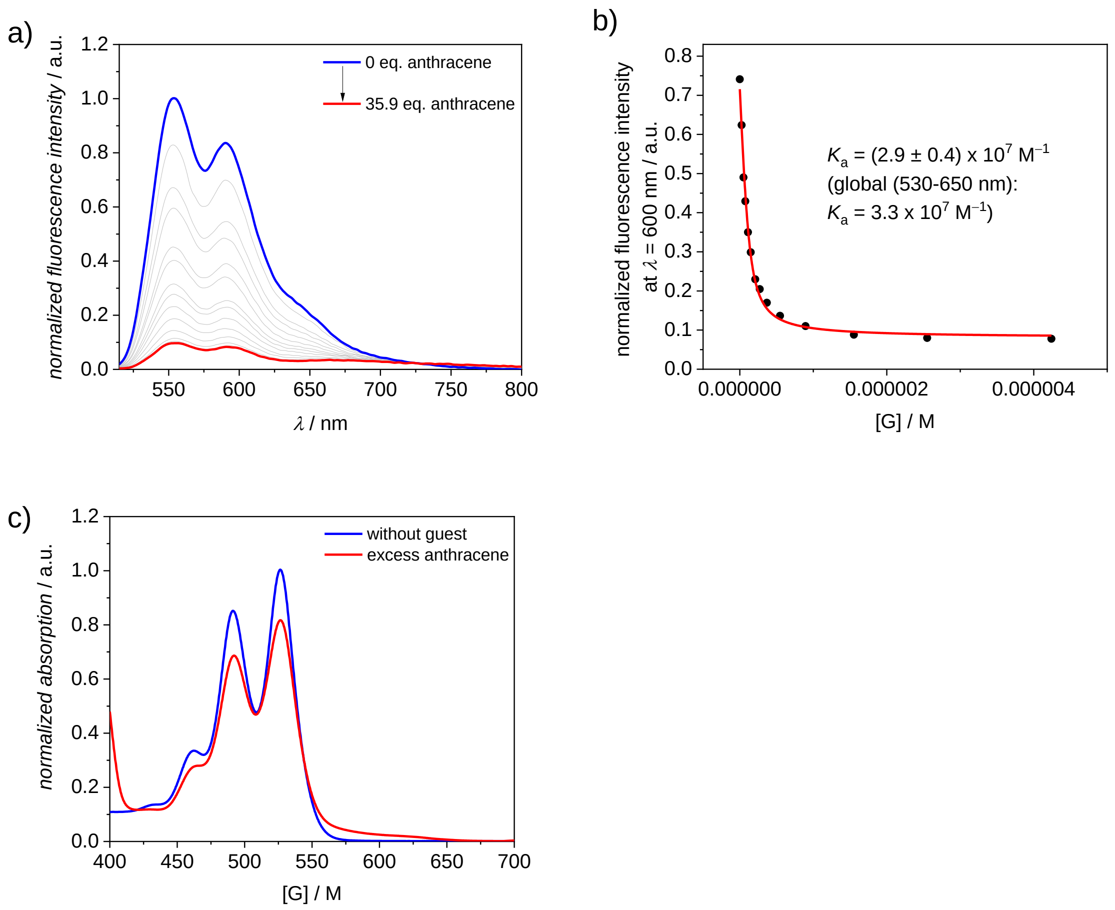

Fig. S8. a) Fluorescence spectra (λ_{exc} = 507 nm) of cyclophane **1-*PP*** in CCl_4 at 22 °C (c = 1.2 x 10^{-7} M) upon the addition of anthracene as a guest and b) the resulting plot of the fluorescence at λ = 600 nm with nonlinear curve fit (1:1 binding model, red curve). c) UV/vis spectrum of free **1-*PP*** and anthracene⊂**1-*PP*** in tetrachloromethane at 22 °C.

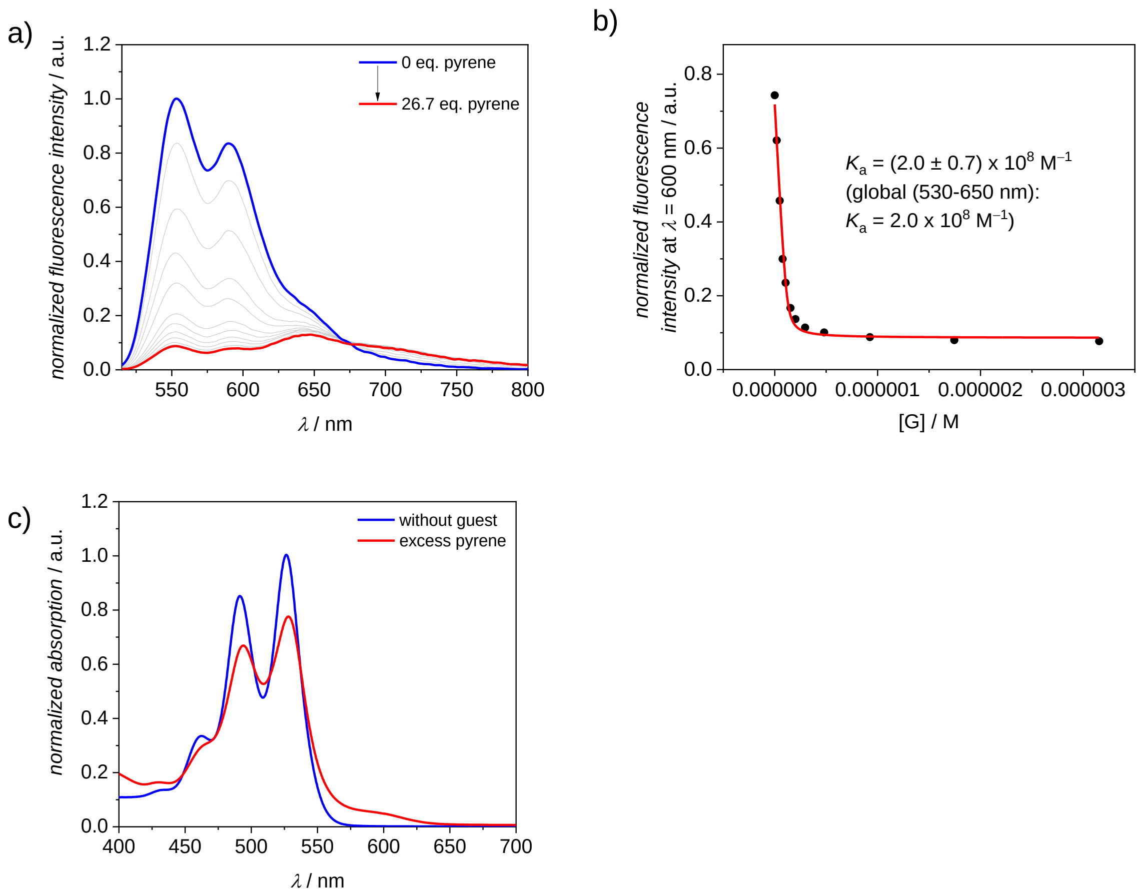

Fig. S9. a) Fluorescence spectra (λ_{exc} = 503 nm) of cyclophane **1-*PP*** in CCl_4 at 22 °C (c = 1.2 x 10^{-7} M) upon the addition of pyrene as a guest and b) the resulting plot of the fluorescence at λ = 600 nm with nonlinear curve fit (1:1 binding model, red curve). c) UV/vis spectrum of free **1-*PP*** and pyrene⊂**1-*PP*** in tetrachloromethane at 22 °C.

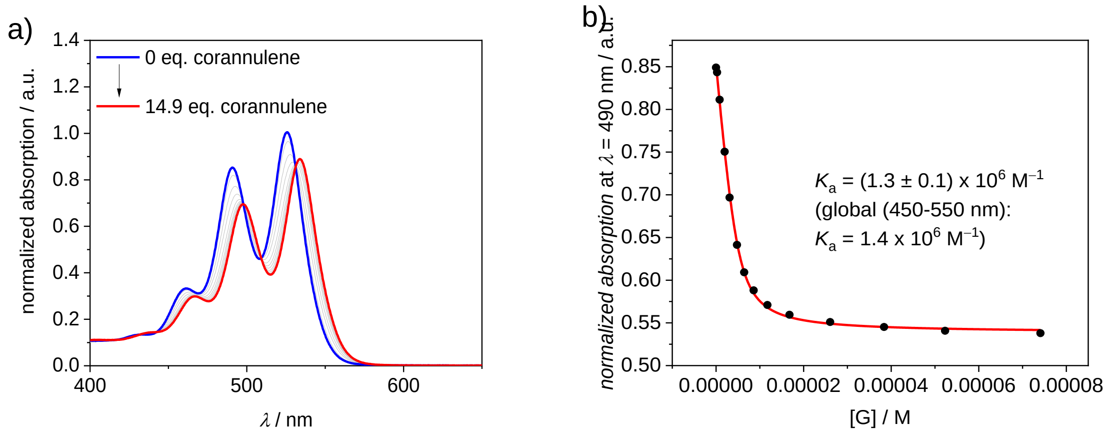

Fig. S10. a) UV/vis spectra of cyclophane **1-*PP*** in CCl_4 at 22 °C (c = 5.0 x 10^{-6} M) upon the addition of corannulene as a guest and b) the resulting plot of the absorption at λ = 490 nm with nonlinear curve fit (1:1 binding model, red curve).

4. Titration Studies in Dichloromethane

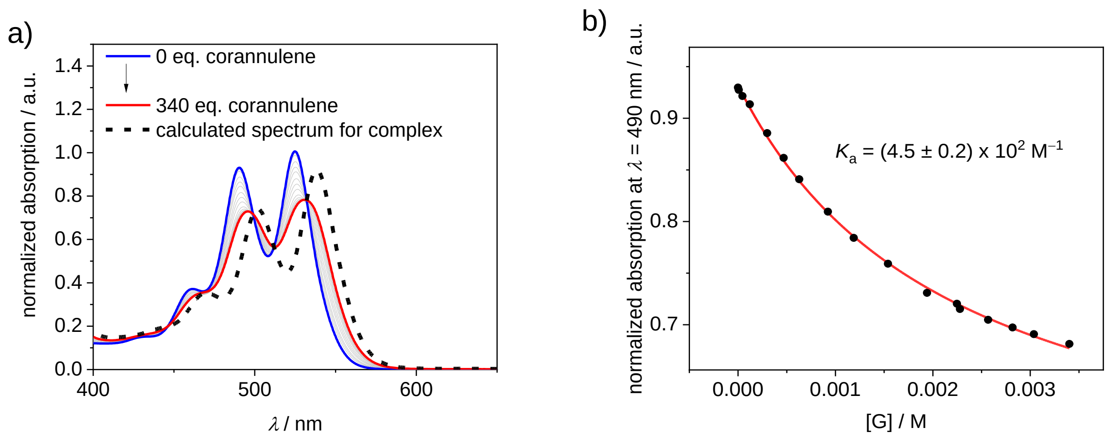

Fig. S11. a) UV/vis spectra of cyclophane **1-*PP*** in CH_2Cl_2 at 22 °C ($c = 10 \times 10^{-6}$ M) upon the addition of corannulene as a guest and b) the resulting plot of the absorption at λ = 490 nm with nonlinear curve fit (1:1 binding model, red curve). The spectrum of the 1:1 complex was calculated from the titration data.

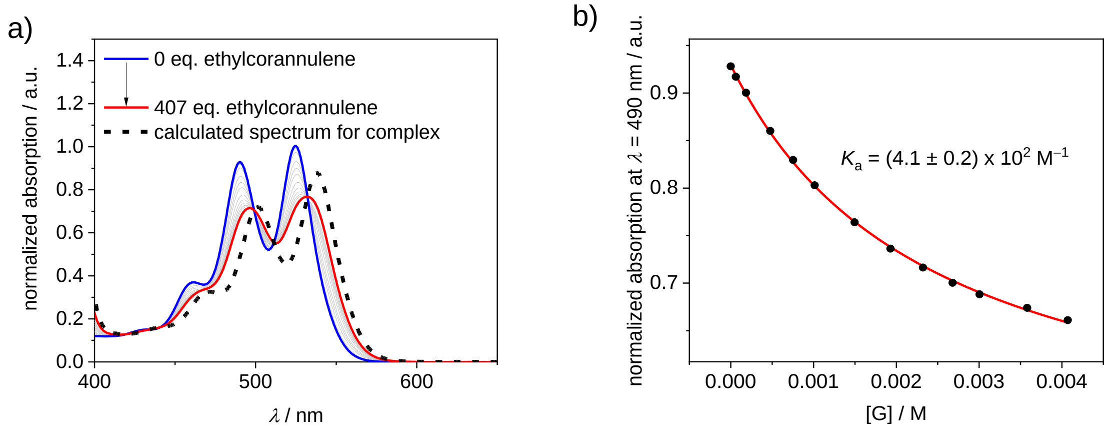

Fig. S12. a) UV/vis spectra of cyclophane **1-*PP*** in CH_2Cl_2 at 22 °C ($c = 10 \times 10^{-6}$ M) upon the addition of ethylcorannulene as a guest and b) the resulting plot of the absorption at λ = 490 nm with nonlinear curve fit (1:1 binding model, red curve). The spectrum of the 1:1 complex was calculated from the titration data.

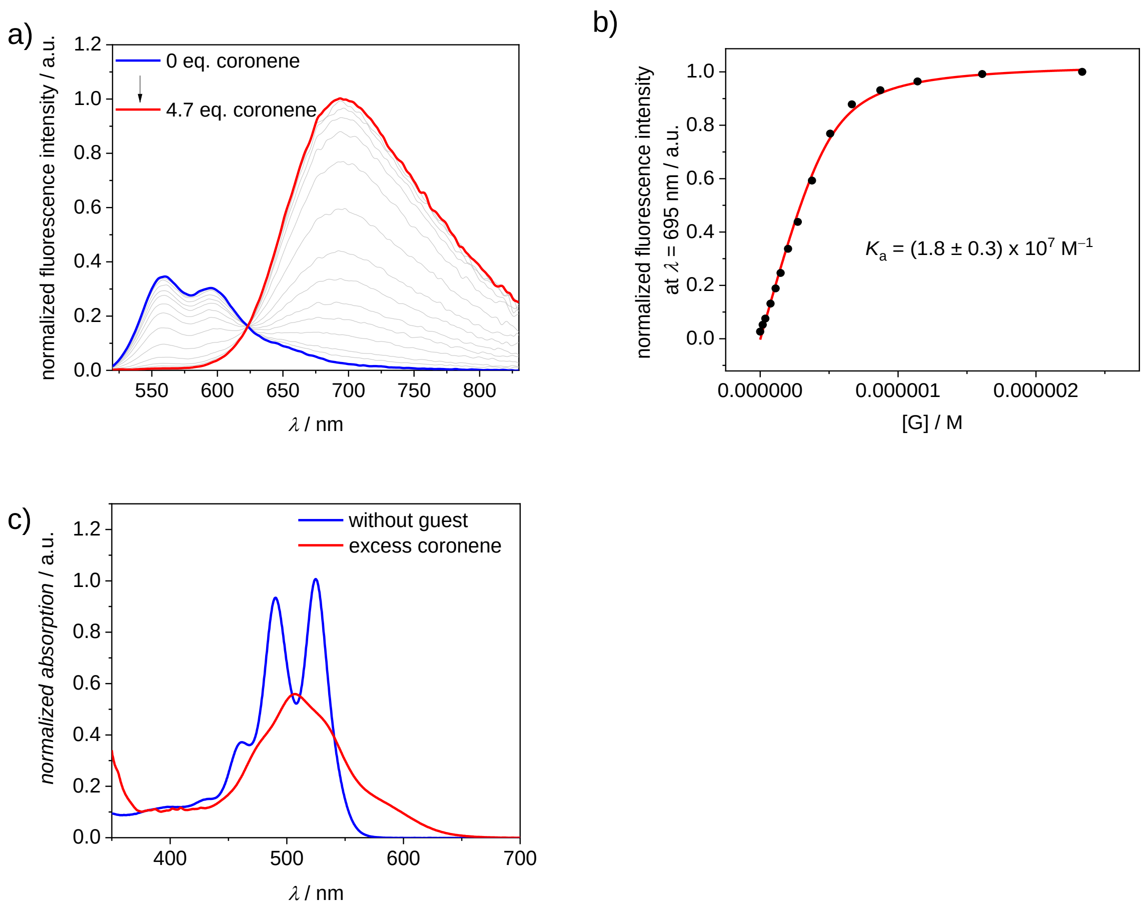

Fig. S13. Fluorescence spectra (λ_{exc} = 505 nm) of cyclophane **1-*PP*** in CH_2Cl_2 at 22°C (c = 5.0 x 10^{-7} M) upon the addition of coronene as a guest and the resulting plot of the absorption at λ = 695 nm with nonlinear curve fit (1:1 binding model, red curve). c) UV/vis spectrum of free **1-*PP*** and coronene⊂**1-*PP*** in dichloromethane at 22 °C.

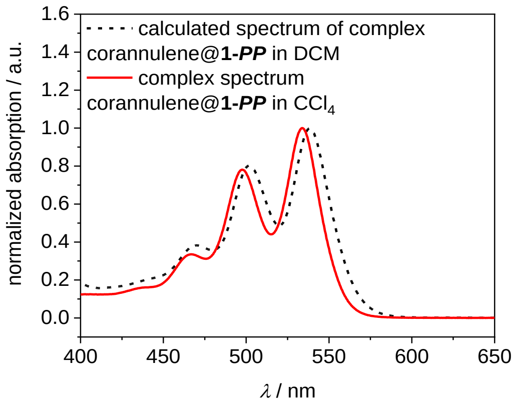

Fig. S14. UV/vis spectrum of corannulene⊂**1-*PP*** in CCl_4 and the corresponding calculated spectrum (the spectrum of the 1:1 complex was calculated from the titration data) in DCM.

5. Variable Temperature NMR Studies

From the coalescence temperature and the signal splitting of the methylene protons at the lowest measured temperature, the corresponding barrier for the bowl-to-bowl inversion can be determined according to equation S1 with the universal gas constant *R*, the coalescence temperature T_c, the Avogadro constant N_A, Planck's constant *h* and the signal splitting at the lowest measured temperature Δν.

$$\Delta G^{\ddagger} = RT_C \ln\left(\frac{RT_C\sqrt{2}}{\pi N_A h \Delta\nu}\right) \tag{S1}$$

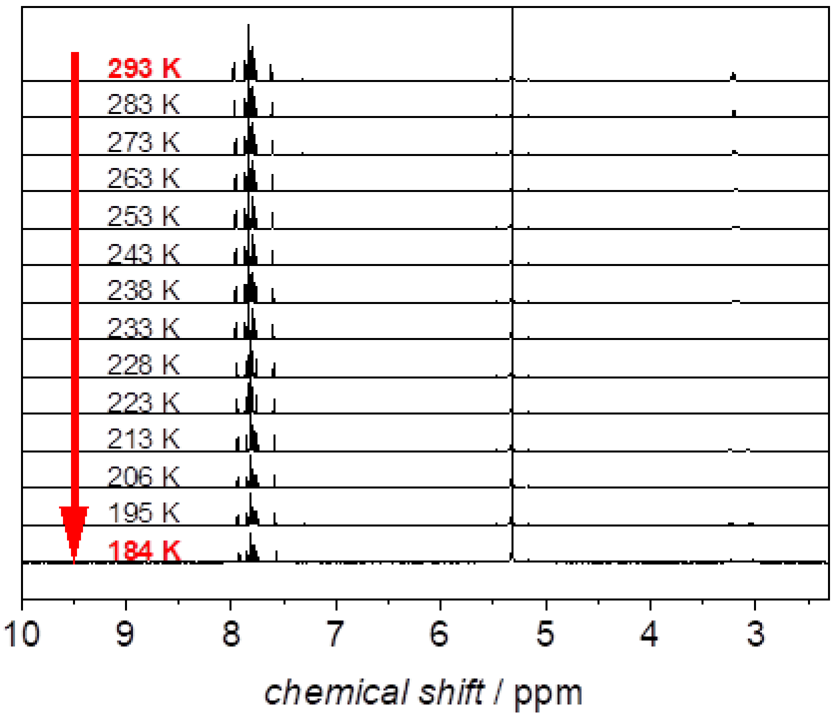

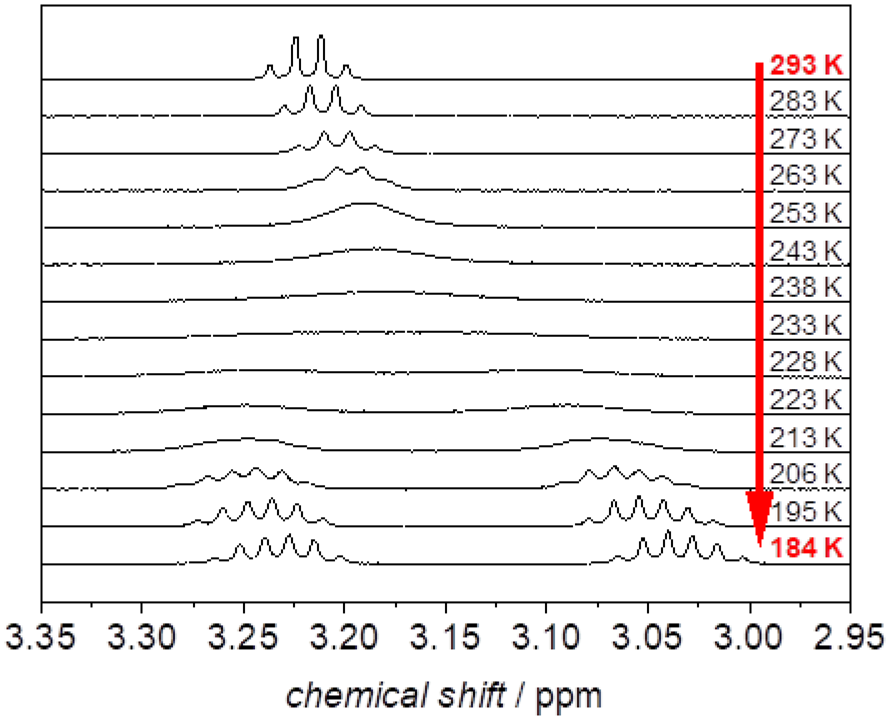

Fig. S15. Variable-temperature NMR (600 MHz) study of ethylcorannulene in DCM-d_2 (c ≈ 1 mM).

Tc = (233 ± 2) K

Δν = (120 ± 5) Hz

$\Delta G^{\ddagger}$= (45.8 ± 0.5) kJ mol^{-1}

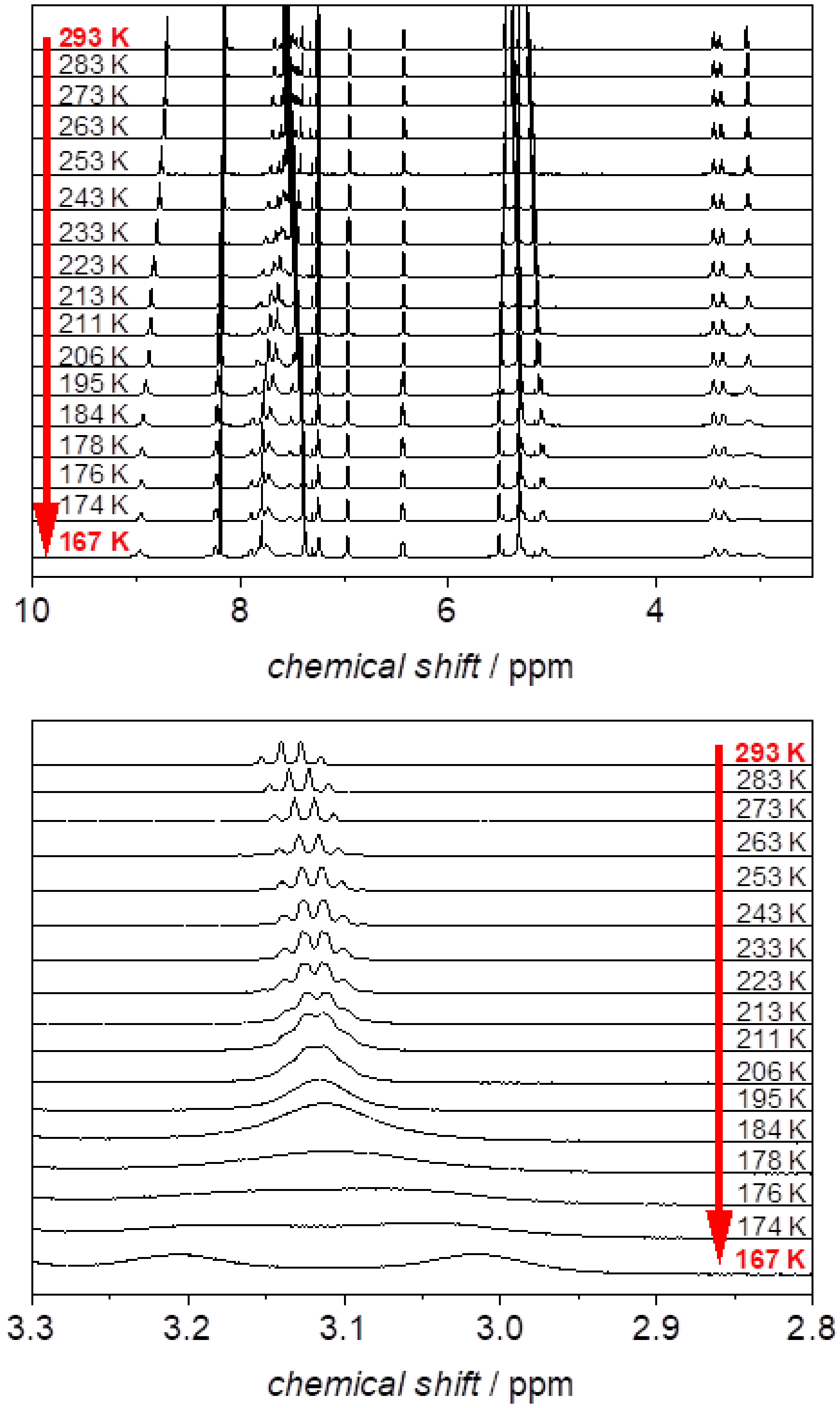

Fig. S16. Variable-temperature NMR (600 MHz) study of ethylcorannulene in the presence of 0.5 equivalents of **1-*PP*** in DCM-d_2 (c (**1-*PP***) ≈ 1 mM). At room temperature, 20% of guest are bound under these conditions.

Tc = (176 ± 2) K

Δν = (116 ± 5) Hz

$\Delta G^{\ddagger}$= (34.2 ± 0.4) kJ mol^{-1}

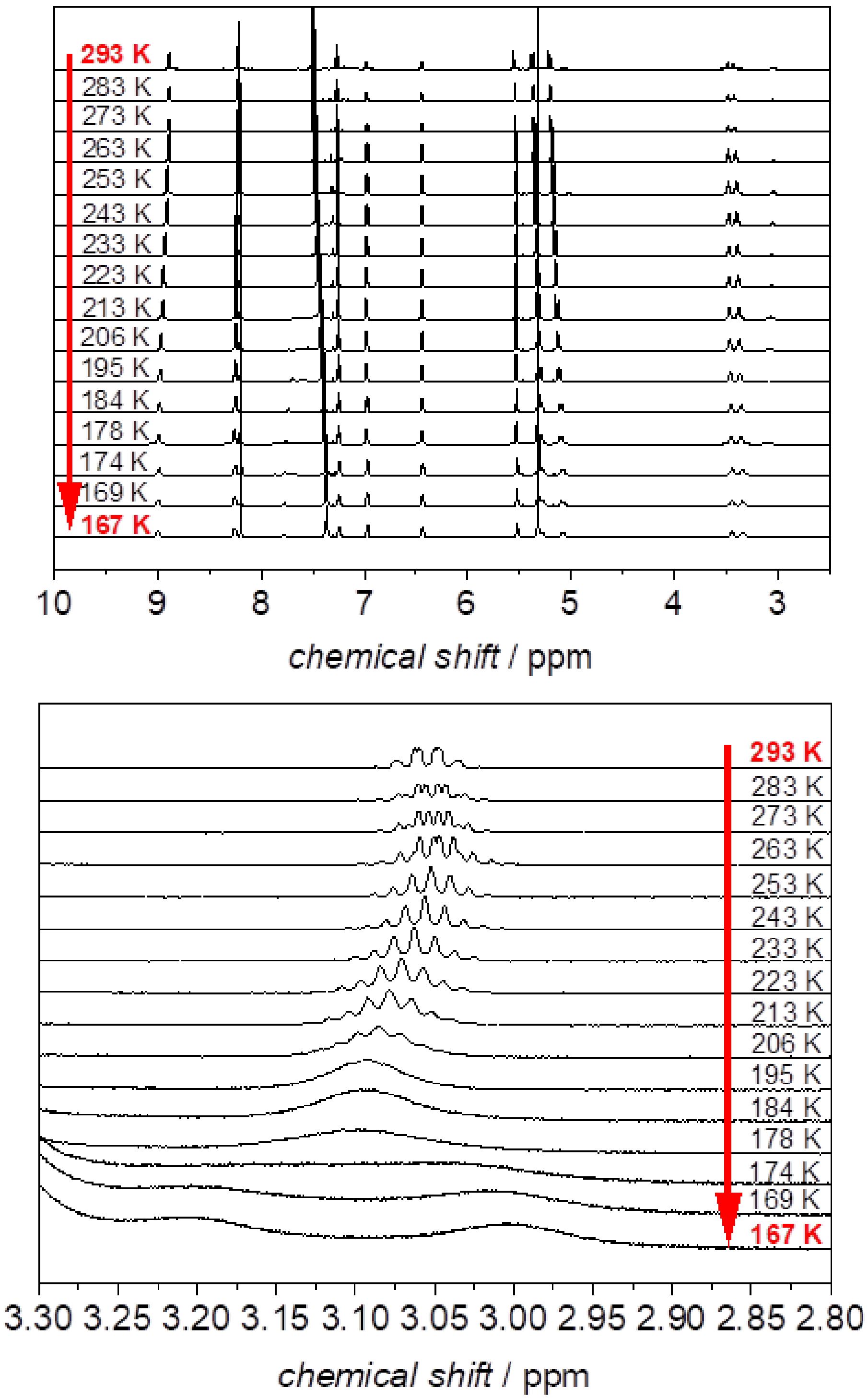

Fig. S17. Variable-temperature NMR (600 MHz) study of ethylcorannulene in the presence of two equivalents of **1-*PP*** in DCM-d_2 (c (**1-*PP***) ≈ 2 mM). At room temperature, 40% of guest are bound under these conditions.

Tc = (174 ± 2) K

Δν = (122 ± 5) Hz

Δ$G^‡$= (33.7 ± 0.4) kJ mol^{-1}

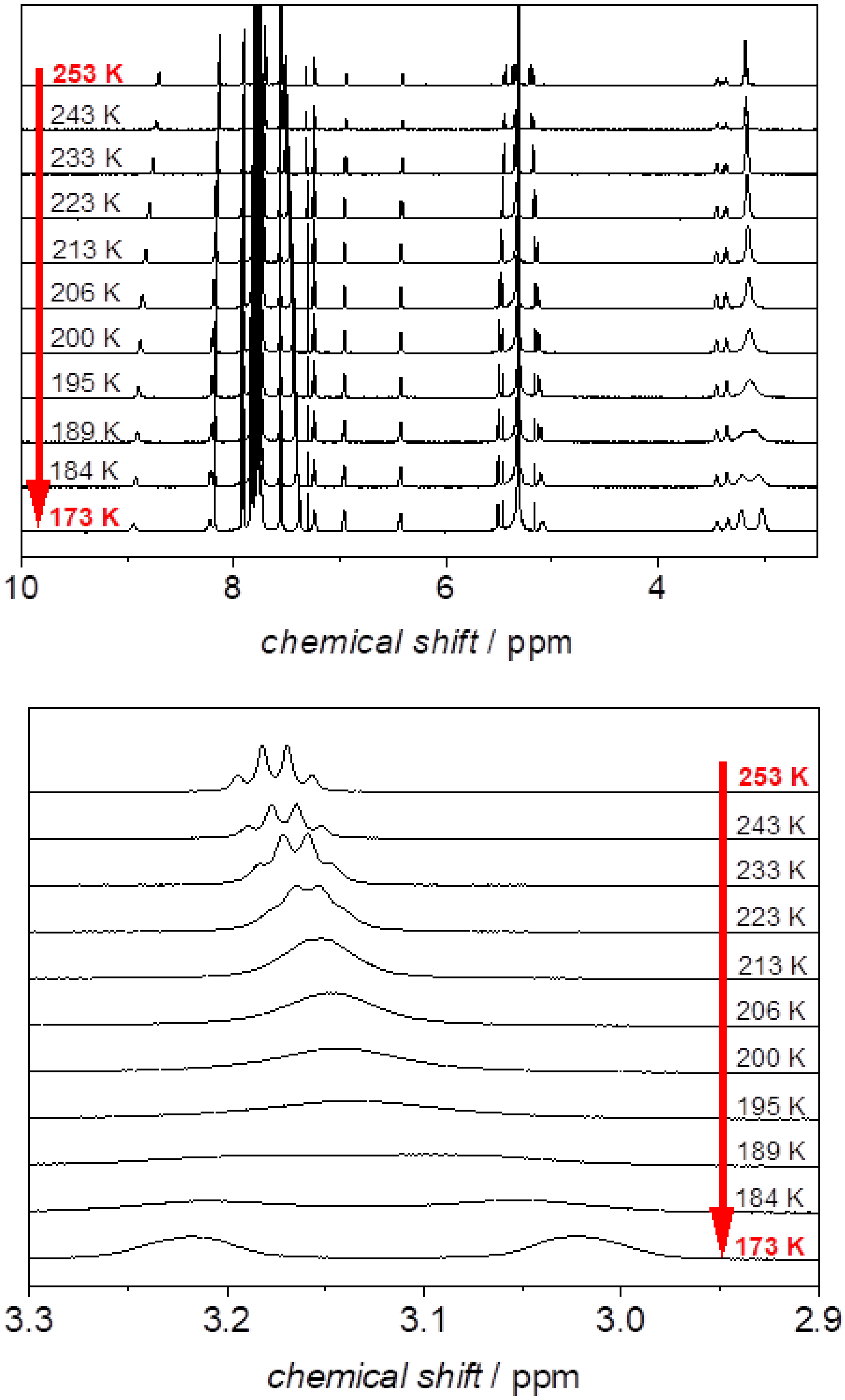

Fig. S18. Variable-temperature NMR (600 MHz) study of ethylcorannulene in the presence of 0.1 equivalents of **1-*PP*** in DCM-d_2 (c (**1-*PP***) ≈ 0.2 mM). At room temperature, 4% of guest are bound under these conditions.

Tc = (189 ± 2) K

Δν = (117 ± 5) Hz

$\Delta G^{\ddagger}$= (36.8 ± 0.5) kJ mol^{-1}

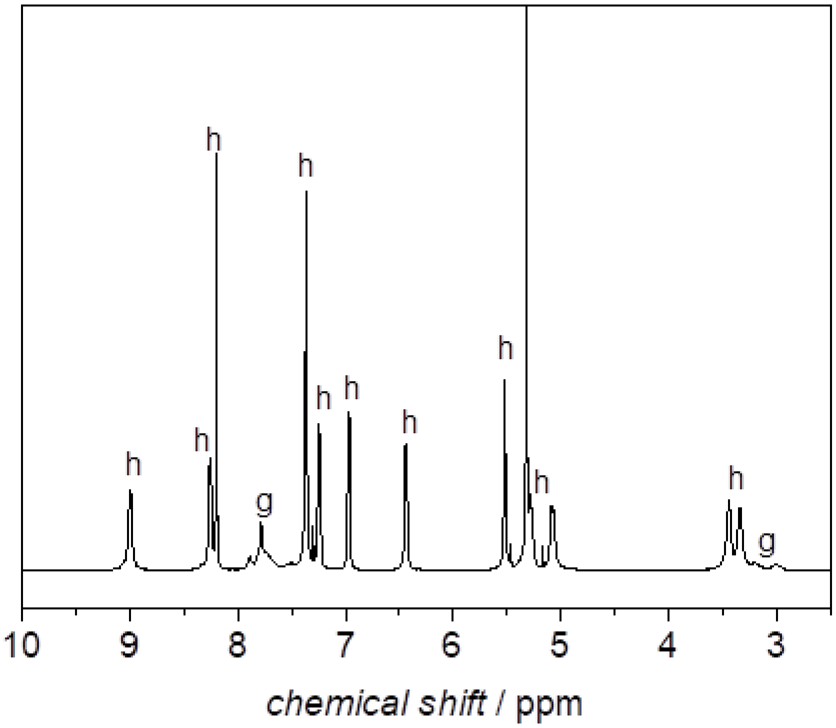

Fig. S19. Low-temperature NMR (167 K, 600 MHz) of ethylcorannulene in the presence of two equivalents of a) **1-*PP*** ($c \approx 2$ mM). One set of host protons are visible (signals of host are marked with “h” and signals of guest with “g”).

6. Single Crystal X-Ray Analysis

The crystals of coronene⊂**1-*PP*** were grown from a host-guest mixture in chlorobenzene by slow evaporation of *n*-hexane into the solution and were obtained as red blocks.

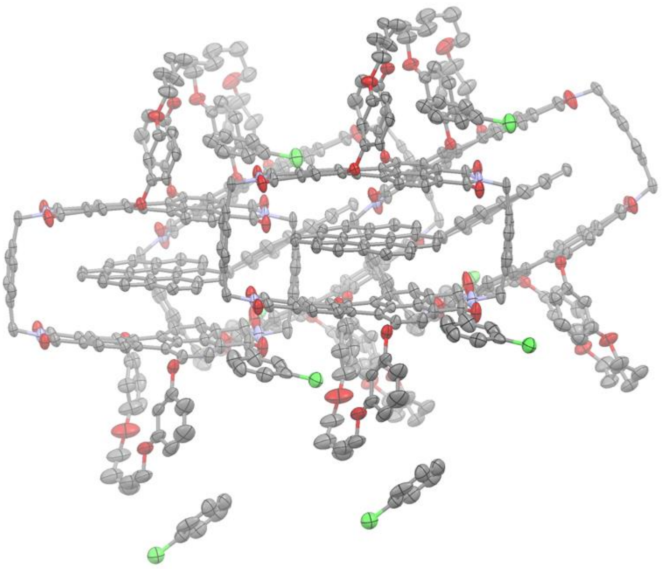

Fig. S20. Packing of coronene⊂**1-*PP*** in the crystalline state, obtained by single crystal X-ray analysis (thermal ellipsoids set at 50% probability). Hydrogen atoms are omitted for clarity.

Table S1. Crystal data and structure refinement for coronene⊂**1-*PP***.

Identification code	coronene⊂**1-*PP***
CCDC number	2300009
Empirical formula	$C_{136}H_{90}Cl_2N_4O_{16}$
Formula weight	2107.01
Temperature	100(2) K
Wavelength	1.54178 Å
Crystal system	Triclinic
Space group	*P1*
Unit cell dimensions	a = 13.2705 (10) Å
	b = 21.6957 (16) Å
	c = 22.1130 (17) Å
Volume	5394.3 (7) $Å^3$
Z	2
Density (calculated)	1.297 g/cm^3
Absorption coefficient	1.124 mm^{-1}
F(000)	2192
Crystal size	0.256 × 0.190 × 0.095 mm^3
Theta range for data collection	2.280 to 72.599°
Index ranges	$-16 \le h \le 16$, $-26 \le k \le 26$, $-27 \le l \le 27$
Reflections collected	435202
Independent reflections	41430 [R(int) = 0.0355]
Completeness to theta = 67.679°	100%
Absorption correction	Semi-empirical from equivalents
Refinement method	Full-matrix least-squares on F^2
Data / restraints / parameters	41430 / 2756 / 3240
Goodness-of-fit on F^2	1.015
Final R indices [$I > 2\sigma(I)$]	R_1 = 0.0503, wR_2 = 0.1461
R indices (all data)	R_1 = 0.0515, wR_2 = 0.1478
Absolute structure parameter	0.053 (3)
Largest diff. peak and hole	1.087 and -0.589 e·$Å^{-3}$

7. DFT Calculations

Table S2. Core twist of the calculated complex structures with corannulene as a guest.

structure	average core twist (and core twist of the two chromophores)
**1-*PP***	18.1° (17.4° and 18.7°)
corannulene⊂**1-*PP***	15.4° (12.3° and 18.5°)
[corannulene⊂**1-*PP***]‡	11.6° (12.4° and 10.7°)

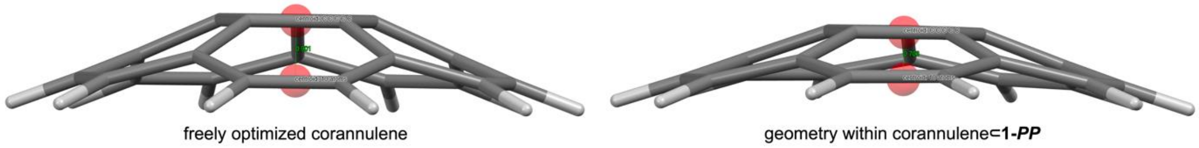

Fig. S21. Comparison between the bowl-depths of the freely optimized corannulene and the compressed corannulene from the optimized complex characterized by the distance between the centroids of the central 5-membered ring and the 10 carbon atoms making up the corannulene rim.

Table S3. Breakdowns of the intermolecular interaction energies (ΔE_{INT}) in kJ mol^{-1} between host and guest in reactant complex corannulene⊂**1-*PP*** and transition structure complex [corannulene⊂**1-*PP***]‡ from second generation ALMO-EDA at the ωB97MV/Def2-SVP level of theory.

structure	$\Delta E_{\mathrm{PAULI}}$	ΔE_{ELEC}	ΔE_{DISP}	ΔE_{POL}	ΔE_{CT}	ΔE_{INT}
corannulene⊂**1-*PP***	452.6	–286.0	–360.5	–10.7	–15.4	–219.9
[corannulene⊂**1-*PP***]‡	479.0	–307.2	–387.4	–10.7	–14.7	–241.0

Cartesian coordinates, calculated absolute Gibbs free energies and imaginary frequencies for all optimized structures

Cartesian coordinates (in Å) for all optimized structures (B3LYP-D3(BJ)/def2-SVP), calculated absolute energies (SMD-(CCl_4)-PW6B95-D3(BJ)/Def2-TZVPP//B3LYP-D3(BJ)/def2-SVP, in Hartree), number of imaginary frequencies and imaginary frequencies pertaining to the optimized transition structures.

Free equilibrium structures

corannulene (equilibrium structure)

C	1.310892	2.987398	-0.262281
C	2.436097	2.169889	-0.262281
C	-1.310892	2.987398	-0.262281
C	-0.000000	2.488304	0.097767
C	-0.000000	1.209475	0.638881
C	2.366518	0.768928	0.097767
C	-2.436097	2.169889	-0.262281
C	3.246272	-0.323575	-0.262281
C	0.695415	-3.187379	-0.262281
C	-0.695415	-3.187379	-0.262281
C	1.462588	-2.013080	0.097767
C	0.710912	-0.978486	0.638881
C	-0.710912	-0.978486	0.638881
C	-1.462588	-2.013080	0.097767
C	-2.816482	-1.646333	-0.262281
C	-3.246272	-0.323575	-0.262281
C	-2.366518	0.768928	0.097767
C	-1.150279	0.373748	0.638881
C	2.816482	-1.646333	-0.262281
C	1.150279	0.373748	0.638881
H	-4.249855	-0.102081	-0.636258
H	-3.498207	-2.415417	-0.636258
H	-1.216193	-4.073397	-0.636258
H	1.216193	-4.073397	-0.636258
H	3.498207	-2.415417	-0.636258
H	4.249855	-0.102081	-0.636258

H	3.378203	2.580587	-0.636258
H	1.410362	4.010308	-0.636258
H	-1.410362	4.010308	-0.636258
H	-3.378203	2.580587	-0.636258

Number of imaginary frequencies = 0

Computed total Gibbs free energy = -769.30078 a.u.

corannulene (TS)

C	1.368772	3.080237	0.000000
C	2.506506	2.253625	-0.000000
C	-1.368772	3.080237	-0.000000
C	-0.000000	2.561866	0.000000
C	-0.000000	1.192150	0.000000
C	2.436479	0.791660	0.000000
C	-2.506506	2.253625	0.000000
C	3.352453	-0.349934	0.000000
C	0.703158	-3.296508	0.000000
C	-0.703158	-3.296508	-0.000000
C	1.505827	-2.072593	0.000000
C	0.700728	-0.964470	0.000000
C	-0.700728	-0.964470	0.000000
C	-1.505827	-2.072593	0.000000
C	-2.917878	-1.687420	0.000000
C	-3.352453	-0.349934	0.000000
C	-2.436479	0.791660	0.000000
C	-1.133802	0.368395	0.000000
C	2.917878	-1.687420	0.000000
C	1.133802	0.368395	0.000000
H	-3.690451	-2.461436	0.000000
H	-4.432433	-0.177849	-0.000000
H	-3.481377	2.749202	0.000000
H	-1.538841	4.160536	0.000000
H	1.538841	4.160536	0.000000
H	3.481377	2.749202	-0.000000

H	4.432433	-0.177849	0.000000
H	3.690451	-2.461436	0.000000
H	1.200553	-4.270453	0.000000
H	-1.200553	-4.270453	-0.000000

Number of imaginary frequencies = 1 (-114.0*i* cm^{-1})

Computed total Gibbs free energy = -769.28259 a.u.

1-PP

O	3.487096	5.962768	2.139223
O	3.041978	5.712872	-2.394147
O	2.783197	-5.374432	2.664499
O	3.679876	-5.650598	-1.800056
O	-3.679857	-5.650629	1.799779
O	-2.783242	-5.374382	-2.664783
O	-3.041741	5.712831	2.394033
O	-3.487190	5.962806	-2.139300
O	-4.248375	-0.954956	3.399693
O	-8.915799	-0.622089	2.469019
O	-8.441495	-0.066385	-2.588760
O	-4.021716	1.271559	-3.749441
N	3.244143	5.845171	-0.126841
N	3.213799	-5.520217	0.430250
N	-3.213852	-5.520210	-0.430539
N	-3.244074	5.845166	0.126746
C	3.444917	5.274955	1.134252
C	3.573720	3.796690	1.182851
C	3.771573	3.176730	2.402110
H	3.885941	3.774199	3.306693
C	3.806581	1.778029	2.492485
C	3.608260	0.954363	1.367227
C	3.428026	-0.499750	1.432252
C	3.109122	-1.168462	2.620377
H	2.943693	-0.596411	3.526880
C	2.979418	-2.560860	2.673973

H	2.726276	-3.063805	3.608088
C	3.184770	-3.327814	1.539362
C	3.049048	-4.802415	1.622103
C	3.536139	-4.957008	-0.808349
C	3.663221	-3.478713	-0.853408
C	3.951391	-2.863237	-2.056762
H	4.129171	-3.465170	-2.948096
C	3.985315	-1.465110	-2.152681
C	3.720845	-0.631926	-1.049345
C	3.565565	0.827053	-1.131521
C	3.393096	1.507058	-2.344041
H	3.339711	0.942547	-3.267705
C	3.266641	2.900143	-2.401901
H	3.121033	3.408684	-3.355812
C	3.337283	3.660408	-1.246891
C	3.203795	5.135289	-1.333717
C	3.493939	3.016285	0.004058
C	3.557913	1.590380	0.084043
C	3.547045	-1.265733	0.225869
C	3.472278	-2.691193	0.307315
C	-3.536143	-4.957020	0.808082
C	-3.663183	-3.478724	0.853178
C	-3.951258	-2.863265	2.056563
H	-4.128982	-3.465211	2.947899
C	-3.985140	-1.465140	2.152512
C	-3.720754	-0.631940	1.049169
C	-3.565453	0.827035	1.131359
C	-3.392899	1.507018	2.343880
H	-3.339466	0.942490	3.267531
C	-3.266425	2.900100	2.401755
H	-3.120749	3.408624	3.355665
C	-3.337136	3.660385	1.246763
C	-3.203635	5.135264	1.333605
C	-3.444933	5.274974	-1.134344

C	-3.573758	3.796712	-1.182958
C	-3.771736	3.176778	-2.402209
H	-3.886192	3.774266	-3.306768
C	-3.806774	1.778080	-2.492608
C	-3.608337	0.954389	-1.367385
C	-3.428096	-0.499724	-1.432446
C	-3.109230	-1.168417	-2.620592
H	-2.943824	-0.596352	-3.527089
C	-2.979525	-2.560814	-2.674215
H	-2.726411	-3.063743	-3.608346
C	-3.184837	-3.327787	-1.539611
C	-3.049104	-4.802385	-1.622379
C	-3.493888	3.016286	-0.004186
C	-3.557882	1.590383	-0.084193
C	-3.547046	-1.265727	-0.226069
C	-3.472292	-2.691186	-0.307541
C	-5.510340	-0.477283	3.685499
C	-6.616969	-0.757843	2.888870
H	-6.540414	-1.369762	1.991108
C	-7.879167	-0.267887	3.268248
C	-8.010750	0.522421	4.420036
H	-8.975107	0.928171	4.719416
C	-6.877388	0.791656	5.195623
H	-6.981184	1.404875	6.093796
C	-5.625273	0.296611	4.847500
H	-4.739577	0.489538	5.453739
C	-10.238829	-0.350171	2.911291
H	-10.348047	-0.691921	3.956216
H	-10.423811	0.741089	2.896775
C	-11.246892	-1.071615	2.033150
H	-12.213162	-1.009113	2.562152
H	-10.980605	-2.142393	2.001507
C	-11.432425	-0.528018	0.613339
H	-12.376128	-0.940004	0.213322

H	-11.584762	0.566232	0.661552
C	-10.312246	-0.842390	-0.381254
H	-9.368096	-0.377400	-0.060933
H	-10.128265	-1.932485	-0.387684
C	-10.671726	-0.381228	-1.794445
H	-10.817253	0.713006	-1.807565
H	-11.637081	-0.826104	-2.094857
C	-9.658017	-0.743990	-2.862905
H	-10.031941	-0.449004	-3.860254
H	-9.481993	-1.836610	-2.880304
C	-7.419752	-0.158301	-3.470928
C	-6.276414	0.593339	-3.151916
H	-6.280846	1.181967	-2.235939
C	-5.179894	0.565847	-4.006909
C	-5.195320	-0.188604	-5.186345
H	-4.321505	-0.182315	-5.838768
C	-6.334184	-0.930038	-5.486261
H	-6.357979	-1.529925	-6.399000
C	-7.449781	-0.932073	-4.641269
H	-8.321095	-1.530337	-4.900949
C	2.907848	7.283883	-0.177760
H	3.418173	7.760135	0.665586
H	3.298451	7.673438	-1.123417
C	1.407487	7.460999	-0.087522
C	0.621607	7.483490	-1.248745
H	1.109805	7.458638	-2.224679
C	-0.771024	7.485743	-1.162356
H	-1.376452	7.466968	-2.070707
C	-1.407430	7.461003	0.087436
C	-0.621550	7.483547	1.248656
H	-1.109745	7.458737	2.224592
C	0.771081	7.485797	1.162267
H	1.376506	7.467062	2.070621
C	-2.907789	7.283881	0.177676

H	-3.418118	7.760133	-0.665666
H	-3.298392	7.673428	1.123336
C	2.876778	-6.959480	0.456933
H	3.467102	-7.439190	-0.330384
H	3.174440	-7.342999	1.438214
C	1.392423	-7.139614	0.222812
C	0.879588	-7.165179	-1.082565
H	1.569835	-7.146812	-1.928271
C	-0.498182	-7.162605	-1.303027
H	-0.889839	-7.137136	-2.321503
C	-1.392471	-7.139610	-0.223146
C	-0.879636	-7.165266	1.082229
H	-1.569882	-7.146967	1.927936
C	0.498134	-7.162695	1.302691
H	0.889791	-7.137298	2.321168
C	-2.876829	-6.959473	-0.457254
H	-3.467149	-7.439203	0.330054
H	-3.174497	-7.342968	-1.438542
O	4.248697	-0.954914	-3.399827
O	8.916009	-0.622054	-2.468604
O	8.441242	-0.066506	2.589139
O	4.021347	1.271466	3.749331
C	5.510685	-0.477198	-3.685459
C	6.617226	-0.757799	-2.888723
H	6.540574	-1.369782	-1.991012
C	7.879463	-0.267807	-3.267926
C	8.011169	0.522578	-4.419648
H	8.975555	0.928356	-4.718893
C	6.877891	0.791854	-5.195344
H	6.981782	1.405133	-6.093465
C	5.625741	0.296772	-4.847396
H	4.740113	0.489730	-5.453724
C	10.239088	-0.350131	-2.910728
H	10.348403	-0.691826	-3.955661

H	10.424085	0.741126	-2.896135
C	11.247053	-1.071636	-2.032525
H	12.213374	-1.009132	-2.561434
H	10.980738	-2.142409	-2.000953
C	11.432465	-0.528103	-0.612674
H	12.376121	-0.940126	-0.212585
H	11.584829	0.566146	-0.660827
C	10.312185	-0.842491	0.381800
H	9.368074	-0.377469	0.061408
H	10.128182	-1.932583	0.388172
C	10.671543	-0.381389	1.795042
H	10.817104	0.712841	1.808212
H	11.636855	-0.826306	2.095532
C	9.657717	-0.744150	2.863391
H	10.031550	-0.449199	3.860785
H	9.481660	-1.836766	2.880747
C	7.419401	-0.158421	3.471193
C	6.276100	0.593222	3.152053
H	6.280634	1.181853	2.236079
C	5.179486	0.565735	4.006924
C	5.194771	-0.188722	5.186358
H	4.320883	-0.182431	5.838683
C	6.333601	-0.930158	5.486402
H	6.357292	-1.530046	6.399143
C	7.449296	-0.932193	4.641538
H	8.320579	-1.530457	4.901317

Number of imaginary frequencies = 0

Computed total Gibbs free energy = -5282.049924 a.u.

Host–guest complexes

corannulene⊂1-PP

O	-3.322613	5.755368	-2.329046
O	-2.836219	5.533164	2.198350

O	-3.144146	-5.621123	-2.781413
O	-3.840510	-5.806948	1.722893
O	3.393173	-5.753119	-1.891281
O	2.482045	-5.420232	2.565146
O	3.043906	5.578034	-2.508738
O	3.363820	5.847339	2.031364
O	4.244770	-1.105428	-3.491676
O	8.903991	-0.869512	-2.490919
O	8.445399	-0.201108	2.576595
O	4.024224	1.189117	3.666204
N	-3.135058	5.660685	-0.057345
N	-3.468850	-5.722826	-0.526495
N	2.954893	-5.595654	0.342192
N	3.237822	5.724495	-0.242299
C	-3.294904	5.075112	-1.317130
C	-3.407209	3.597504	-1.357133
C	-3.613976	2.972190	-2.571591
H	-3.730979	3.564385	-3.479069
C	-3.685314	1.576710	-2.648253
C	-3.504080	0.753640	-1.519018
C	-3.436073	-0.709098	-1.573506
C	-3.258345	-1.409952	-2.772744
H	-3.107883	-0.859170	-3.692775
C	-3.241928	-2.806572	-2.821171
H	-3.088101	-3.332158	-3.764431
C	-3.417332	-3.548904	-1.666006
C	-3.343788	-5.027162	-1.735724
C	-3.705416	-5.130727	0.717630
C	-3.731766	-3.649853	0.751982
C	-3.882985	-3.011258	1.967165
H	-4.034445	-3.595783	2.874619
C	-3.821691	-1.616118	2.050715
C	-3.589151	-0.805388	0.923077
C	-3.393911	0.647402	0.979971

C	-3.217486	1.339751	2.182560
H	-3.140337	0.787218	3.108710
C	-3.116796	2.733020	2.229520
H	-2.987696	3.248361	3.179686
C	-3.181015	3.480406	1.068552
C	-3.042987	4.951058	1.145951
C	-3.328289	2.824162	-0.176113
C	-3.399821	1.398654	-0.244457
C	-3.536469	-1.456365	-0.354154
C	-3.573583	-2.883999	-0.426432
C	3.291302	-5.049610	-0.900374
C	3.497803	-3.583008	-0.946023
C	3.819321	-2.986827	-2.150358
H	3.953593	-3.597667	-3.043057
C	3.948229	-1.595331	-2.242735
C	3.744075	-0.749859	-1.136853
C	3.644677	0.711563	-1.223903
C	3.475731	1.384611	-2.439876
H	3.407945	0.815235	-3.358667
C	3.339799	2.774611	-2.504187
H	3.184612	3.276366	-3.459814
C	3.387900	3.539117	-1.351632
C	3.215652	5.007047	-1.444939
C	3.390147	5.156694	1.026795
C	3.566832	3.686387	1.080060
C	3.748140	3.075997	2.306385
H	3.810515	3.677162	3.213074
C	3.827069	1.681804	2.400635
C	3.675540	0.849141	1.274059
C	3.478009	-0.601250	1.346449
C	3.138438	-1.253111	2.538041
H	2.994217	-0.673972	3.441855
C	2.912504	-2.630786	2.589081
H	2.624635	-3.114148	3.522826

C	3.046918	-3.406065	1.449678
C	2.805328	-4.865298	1.528785
C	3.548236	2.903130	-0.097832
C	3.640162	1.479188	-0.012331
C	3.545291	-1.371848	0.139709
C	3.372091	-2.788168	0.218210
C	5.519613	-0.655137	-3.758513
C	6.608605	-0.958370	-2.945624
H	6.504513	-1.566314	-2.047952
C	7.886215	-0.496110	-3.306574
C	8.052285	0.288077	-4.458243
H	9.029557	0.672326	-4.743835
C	6.936642	0.580407	-5.250904
H	7.067012	1.189530	-6.148441
C	5.669059	0.113935	-4.919884
H	4.796240	0.326038	-5.538269
C	10.237945	-0.638760	-2.922720
H	10.347124	-0.994387	-3.963080
H	10.453605	0.446986	-2.918121
C	11.218430	-1.377811	-2.027853
H	12.187668	-1.358438	-2.554800
H	10.915341	-2.438016	-1.977221
C	11.420226	-0.813924	-0.618194
H	12.349354	-1.249338	-0.208869
H	11.609309	0.273473	-0.687407
C	10.289963	-1.070809	0.381398
H	9.360727	-0.583969	0.049788
H	10.072087	-2.154209	0.412026
C	10.664138	-0.589469	1.784269
H	10.838153	0.500639	1.773839
H	11.618459	-1.052867	2.091985
C	9.644337	-0.903334	2.862461
H	10.028849	-0.597617	3.852704
H	9.440737	-1.990577	2.903155

C	7.415553	-0.265613	3.452398
C	6.282588	0.489991	3.108246
H	6.300441	1.059448	2.180573
C	5.175731	0.485581	3.950312
C	5.173376	-0.247341	5.143656
H	4.291288	-0.224143	5.784492
C	6.303505	-0.990638	5.470266
H	6.313033	-1.573452	6.394314
C	7.428096	-1.017084	4.637457
H	8.291927	-1.617133	4.917331
C	-2.868669	7.114310	-0.007932
H	-3.397543	7.561626	-0.855910
H	-3.282432	7.486709	0.934752
C	-1.379060	7.362699	-0.090581
C	-0.606823	7.483138	1.073375
H	-1.104767	7.507733	2.044100
C	0.787617	7.492419	1.000908
H	1.384166	7.527134	1.914259
C	1.435734	7.379766	-0.237198
C	0.662120	7.332418	-1.406170
H	1.161980	7.246755	-2.372203
C	-0.730036	7.326253	-1.334260
H	-1.326373	7.241152	-2.244242
C	2.931520	7.167930	-0.306631
H	3.440592	7.640703	0.539428
H	3.341554	7.540940	-1.250897
C	-3.136604	-7.163123	-0.534086
H	-3.721894	-7.634807	0.261742
H	-3.435106	-7.559532	-1.509586
C	-1.649569	-7.317336	-0.300411
C	-1.135820	-7.337968	1.004795
H	-1.825609	-7.350549	1.850480
C	0.241173	-7.289655	1.224709
H	0.633512	-7.260489	2.242827

C	1.132994	-7.228799	0.145109
C	0.621320	-7.255652	-1.160893
H	1.311806	-7.207885	-2.005002
C	-0.755683	-7.296080	-1.381137
H	-1.148922	-7.273882	-2.399290
C	2.613460	-7.031153	0.382715
H	3.208069	-7.519876	-0.396115
H	2.906221	-7.404033	1.369708
O	-3.979942	-1.092258	3.311036
O	-8.663040	-0.521504	2.596656
O	-8.422127	0.122195	-2.534970
O	-3.960726	1.069710	-3.895815
C	-5.209307	-0.586349	3.676651
C	-6.359786	-0.783249	2.918047
H	-6.348398	-1.355407	1.991634
C	-7.581220	-0.252921	3.369197
C	-7.629566	0.491000	4.557873
H	-8.560791	0.926715	4.914505
C	-6.454111	0.675310	5.295069
H	-6.493090	1.252963	6.221566
C	-5.241215	0.141469	4.873335
H	-4.322248	0.270876	5.446177
C	-9.952817	-0.200553	3.099320
H	-10.042285	-0.579332	4.133336
H	-10.079717	0.898515	3.135442
C	-11.030530	-0.831653	2.234385
H	-11.970241	-0.740223	2.805387
H	-10.824430	-1.912857	2.150721
C	-11.245109	-0.222450	0.845176
H	-12.234979	-0.551694	0.481817
H	-11.315961	0.877163	0.938375
C	-10.204296	-0.577720	-0.219942
H	-9.214035	-0.187594	0.059179
H	-10.096711	-1.676913	-0.268686

C	-10.611895	-0.047944	-1.596244
H	-10.671733	1.054120	-1.572723
H	-11.626439	-0.407947	-1.843660
C	-9.701600	-0.455534	-2.739442
H	-10.118686	-0.106729	-3.701831
H	-9.610482	-1.557348	-2.793371
C	-7.464451	-0.036021	-3.477864
C	-6.242464	0.600946	-3.206186
H	-6.147024	1.160651	-2.277615
C	-5.196252	0.493702	-4.116318
C	-5.342073	-0.226059	-5.308704
H	-4.504960	-0.286069	-6.005162
C	-6.559702	-0.850086	-5.563791
H	-6.685728	-1.420856	-6.486804
C	-7.627008	-0.772076	-4.661266
H	-8.562283	-1.280988	-4.886998
C	-0.066796	3.145525	2.667929
C	-0.162853	2.008788	3.474125
C	-0.011645	3.982555	0.138897
C	0.209252	3.063982	1.244124
C	0.559400	1.794010	0.833315
C	0.014909	0.661394	2.954672
C	-0.050304	3.556429	-1.190030
C	-0.391834	-0.652518	3.430380
C	-0.327755	-2.606320	0.066292
C	-0.251076	-2.171930	-1.257864
C	-0.080749	-1.718894	1.191591
C	0.431685	-0.493673	0.812326
C	0.489274	-0.046814	-0.529859
C	0.060394	-0.794802	-1.608448
C	-0.184579	0.010506	-2.793529
C	-0.156183	1.408016	-2.771959
C	0.146003	2.166524	-1.569821
C	0.543616	1.367858	-0.515436

C	-0.436424	-1.775004	2.598739
C	0.478237	0.643105	1.654152
H	-0.435502	1.939320	-3.685527
H	-0.477908	-0.480239	-3.724595
H	-0.509885	-2.884646	-2.042906
H	-0.643561	-3.636535	0.250030
H	-0.817766	-2.709658	3.017403
H	-0.748363	-0.766466	4.457596
H	-0.461693	2.147030	4.517064
H	-0.302845	4.116501	3.109898
H	-0.213348	5.032862	0.344499
H	-0.287510	4.300906	-1.953601

Number of imaginary frequencies = 0

Computed total Gibbs free energy = -6051.3663 a.u.

[corannulene⊂1-PP]‡

O	3.270674	6.005791	2.324774
O	3.040424	5.801480	-2.225828
O	2.855049	-5.347641	2.818802
O	3.522214	-5.581222	-1.685352
O	-3.463447	-5.577542	1.981187
O	-2.572135	-5.381177	-2.485833
O	-3.139296	5.805443	2.378132
O	-3.311694	5.956353	-2.177034
O	-4.056689	-0.872570	3.498978
O	-8.711195	-0.674096	2.465356
O	-8.166811	-0.017655	-2.642226
O	-3.704005	1.241603	-3.720140
N	3.159296	5.913217	0.048202
N	3.223667	-5.475675	0.572940
N	-3.058881	-5.492008	-0.262163
N	-3.232079	5.890229	0.101709
C	3.268438	5.325938	1.312703
C	3.353612	3.847091	1.353334

C	3.494087	3.217533	2.575082
H	3.591955	3.807398	3.486378
C	3.526715	1.821065	2.652645
C	3.352585	1.004089	1.517021
C	3.229367	-0.454117	1.572808
C	2.994048	-1.140400	2.769812
H	2.819138	-0.579634	3.679042
C	2.955606	-2.535559	2.829522
H	2.769098	-3.050342	3.772534
C	3.146189	-3.288276	1.685131
C	3.070277	-4.764264	1.769345
C	3.425138	-4.894260	-0.682125
C	3.486331	-3.416308	-0.726940
C	3.671814	-2.792995	-1.943937
H	3.808913	-3.387862	-2.846293
C	3.669620	-1.397715	-2.036952
C	3.458083	-0.568029	-0.919458
C	3.352574	0.895498	-0.985415
C	3.282226	1.595678	-2.194665
H	3.236271	1.049230	-3.125679
C	3.222797	2.991206	-2.245154
H	3.150759	3.510018	-3.201224
C	3.243973	3.736866	-1.081823
C	3.149226	5.212227	-1.163794
C	3.310244	3.076269	0.168442
C	3.330517	1.648803	0.238277
C	3.340292	-1.210028	0.359515
C	3.334481	-2.637036	0.442643
C	-3.337471	-4.901891	0.973412
C	-3.448184	-3.424788	0.994497
C	-3.717256	-2.790729	2.190976
H	-3.878152	-3.377504	3.095220
C	-3.772996	-1.393862	2.259237
C	-3.540629	-0.576433	1.136837

C	-3.449159	0.886902	1.188777
C	-3.369089	1.597527	2.393287
H	-3.328892	1.054458	3.328219
C	-3.305106	2.993340	2.429999
H	-3.233146	3.524481	3.379839
C	-3.330409	3.726930	1.257315
C	-3.235985	5.203342	1.322316
C	-3.321035	5.288406	-1.157103
C	-3.400810	3.808862	-1.181435
C	-3.507701	3.164123	-2.398689
H	-3.575752	3.742991	-3.319531
C	-3.532235	1.766906	-2.462772
C	-3.391737	0.964134	-1.313067
C	-3.235644	-0.491564	-1.347014
C	-2.939141	-1.186999	-2.525609
H	-2.752734	-0.635032	-3.437777
C	-2.833254	-2.578326	-2.558839
H	-2.592011	-3.097027	-3.485788
C	-3.018964	-3.320512	-1.405550
C	-2.867428	-4.791066	-1.460044
C	-3.385373	3.053273	0.014267
C	-3.407859	1.624785	-0.040947
C	-3.357196	-1.232552	-0.126366
C	-3.282816	-2.659144	-0.183124
C	-5.343260	-0.460839	3.774387
C	-6.421304	-0.753098	2.943229
H	-6.302868	-1.329345	2.026894
C	-7.707774	-0.316636	3.305201
C	-7.894241	0.429548	4.478645
H	-8.878547	0.793252	4.766834
C	-6.789141	0.711051	5.290163
H	-6.935296	1.290557	6.204724
C	-5.512723	0.271023	4.957036
H	-4.647705	0.475996	5.588793

C	-10.052482	-0.450579	2.877142
H	-10.183412	-0.832834	3.905503
H	-10.264373	0.635741	2.896647
C	-11.018034	-1.162600	1.945194
H	-11.998586	-1.143162	2.450767
H	-10.724788	-2.224862	1.881271
C	-11.183199	-0.571332	0.541614
H	-12.117518	-0.975618	0.112766
H	-11.344506	0.519533	0.624434
C	-10.047300	-0.845060	-0.447647
H	-9.110901	-0.382057	-0.101987
H	-9.854140	-1.932948	-0.484801
C	-10.397021	-0.345514	-1.850850
H	-10.536969	0.749423	-1.836941
H	-11.363990	-0.777390	-2.164907
C	-9.384250	-0.687576	-2.927298
H	-9.759636	-0.372766	-3.918213
H	-9.210272	-1.780003	-2.966635
C	-7.138249	-0.112759	-3.516852
C	-5.986785	0.614707	-3.173802
H	-5.993599	1.188387	-2.248783
C	-4.876726	0.575154	-4.010812
C	-4.889814	-0.164699	-5.199794
H	-4.004531	-0.170729	-5.836634
C	-6.039016	-0.878214	-5.526550
H	-6.061081	-1.465665	-6.447450
C	-7.167293	-0.869643	-4.698010
H	-8.045940	-1.447785	-4.977918
C	2.865517	7.359958	-0.004156
H	3.354488	7.818303	0.861436
H	3.299464	7.745423	-0.932326
C	1.367248	7.561571	0.032110
C	0.625422	7.586039	-1.157530
H	1.149903	7.563154	-2.114375

C	-0.769180	7.582966	-1.122239
H	-1.341513	7.560555	-2.051273
C	-1.449648	7.550529	0.103822
C	-0.707983	7.577997	1.293611
H	-1.232312	7.549053	2.250420
C	0.686482	7.585856	1.258322
H	1.258998	7.566007	2.187388
C	-2.946550	7.339094	0.140261
H	-3.437005	7.786695	-0.730165
H	-3.384239	7.730343	1.064185
C	2.962636	-6.929743	0.607201
H	3.565477	-7.384748	-0.185233
H	3.288273	-7.292365	1.587356
C	1.485563	-7.169526	0.389271
C	0.965793	-7.278647	-0.908603
H	1.650940	-7.307248	-1.757382
C	-0.413837	-7.274860	-1.120938
H	-0.811764	-7.297752	-2.136851
C	-1.299441	-7.168495	-0.039992
C	-0.779864	-7.132155	1.262912
H	-1.465533	-7.055485	2.108342
C	0.598118	-7.129628	1.474892
H	0.997552	-7.046259	2.486984
C	-2.775835	-6.941607	-0.273933
H	-3.381563	-7.390630	0.519993
H	-3.087703	-7.324955	-1.250785
O	3.871211	-0.891804	-3.298884
O	8.569702	-0.627137	-2.515606
O	8.194332	0.143161	2.620877
O	3.760363	1.307843	3.906295
C	5.141310	-0.503593	-3.666994
C	6.261542	-0.744396	-2.876423
H	6.192975	-1.265030	-1.922658
C	7.525460	-0.324264	-3.326280

C	7.648596	0.353779	-4.548346
H	8.614831	0.703808	-4.906197
C	6.502146	0.584365	-5.317941
H	6.598906	1.110763	-6.270408
C	5.246609	0.160409	-4.896411
H	4.348898	0.327314	-5.492839
C	9.888420	-0.395533	-2.989746
H	9.990650	-0.825600	-4.002404
H	10.072990	0.692985	-3.070405
C	10.904432	-1.039272	-2.062070
H	11.867666	-1.008429	-2.599396
H	10.648915	-2.107120	-1.948259
C	11.093206	-0.389814	-0.687110
H	12.059140	-0.738541	-0.280149
H	11.203779	0.703170	-0.813316
C	10.007788	-0.676737	0.354282
H	9.042007	-0.256932	0.035274
H	9.856710	-1.769482	0.426939
C	10.395529	-0.128735	1.729342
H	10.491817	0.970052	1.684426
H	11.391252	-0.515470	2.010708
C	9.445168	-0.482726	2.857802
H	9.856170	-0.138179	3.824393
H	9.309214	-1.579264	2.923629
C	7.214099	0.035045	3.548148
C	6.029430	0.730480	3.255659
H	5.977190	1.292676	2.325190
C	4.963125	0.674230	4.147192
C	5.053899	-0.050414	5.342041
H	4.202258	-0.070379	6.022965
C	6.235721	-0.731269	5.618917
H	6.318203	-1.306311	6.544206
C	7.320964	-0.705669	4.735049
H	8.226486	-1.258877	4.977169

C	-0.026280	3.098024	-0.051465
C	-0.099478	2.640265	1.276415
C	0.146109	2.295053	-2.657291
C	0.014858	2.199979	-1.204876
C	-0.043538	0.889065	-0.807997
C	-0.157812	1.222419	1.634032
C	0.265918	1.170954	-3.495265
C	-0.219829	0.399207	2.841843
C	-0.038499	-3.199990	1.187527
C	0.120512	-3.612049	-0.148061
C	-0.142600	-1.795100	1.587047
C	-0.114784	-0.971706	0.493022
C	0.031565	-1.382093	-0.839293
C	0.188240	-2.673256	-1.268032
C	0.396644	-2.715669	-2.715125
C	0.431923	-1.564329	-3.523286
C	0.264762	-0.207204	-2.999407
C	0.070478	-0.232732	-1.644070
C	-0.222759	-1.008279	2.819007
C	-0.138596	0.433427	0.514042
H	0.537637	-3.680866	-3.207747
H	0.606914	-1.713728	-4.591885
H	0.379271	1.360629	-4.566054
H	0.178606	3.279362	-3.130922
H	0.012176	4.180779	-0.202984
H	-0.112040	3.400044	2.061311
H	-0.261109	0.880545	3.822404
H	-0.269367	-1.520306	3.783575
H	-0.064239	-3.987319	1.944690
H	0.203006	-4.684865	-0.330635

Number of imaginary frequencies = 1 (-59.7*i* cm^{-1})

Computed total Gibbs free energy = -6051.3582 a.u.

8. References

1. M. Weh, J. Rühe, B. Herbert, A.-M. Krause and F. Würthner, *Angew. Chem. Int. Ed.*, 2021, **60**, 15323–15327.
2. C. S. Jones, E. Elliott and J. S. Siegel, *Synlett*, 2004, **2004**, 187−191.
3. M. Juríček, N. L. Strutt, J. C. Barnes, A. M. Butterfield, E. J. Dale, K. K. Baldridge, J. F. Stoddart and J. S. Siegel, *Nat. Chem.*, 2014, **6**, 222−228.
4. A. Spek, *Acta Crystallogr. A*, 1990, **46**, C34.
5. A. D. Becke, *J. Chem. Phys.*, 1993, **98**, 5648−5652.
6. C. Lee, W. Yang and R. G. Parr, *Phys. Rev. B*, 1988, **37**, 785−789.
7. S. H. Vosko, L. Wilk and M. Nusair, *Can. J. Phys.*, 1980, **58**, 1200−1211.
8. P. J. Stephens, F. J. Devlin, C. F. Chabalowski and M. J. Frisch, *J. Phys. Chem.* , 1994, **98**, 11623−11627.
9. S. Grimme, J. Antony, S. Ehrlich and H. Krieg, *J. Chem. Phys.*, 2010, **132**, 154104.
10. S. Grimme, S. Ehrlich and L. Goerigk, *J. Comput. Chem.*, 2011, **32**, 1456−1465.
11. F. Weigend and R. Ahlrichs, *Phys. Chem. Chem. Phys.*, 2005, **7**, 3297−3305.
12. C. Gonzalez and H. B. Schlegel, *J. Chem. Phys.*, 1989, **90**, 2154–2161.
13. C. Gonzalez and H. B. Schlegel, *J. Phys. Chem.*, 1990, **94**, 5523–5527.
14. Y. Zhao and D. G. Truhlar, *J. Phys. Chem. A*, 2005, **109**, 5656−5667.
15. A. V. Marenich, C. J. Cramer and D. G. Truhlar, *J. Phys. Chem. B*, 2009, **113**, 6378−6396.
16. P. R. Horn, Y. Mao and M. Head-Gordon, *Phys. Chem. Chem. Phys.*, 2016, **18**, 23067−23079.
17. M. J. Frisch, 2016, et al. Wallingford, CT.
18. M. Sapotta, A. Hofmann, D. Bialas and F. Würthner, *Angew. Chem. Int. Ed.*, 2019, **58**, 3516–3520.
19. Bindfit, available free of charge via http://supramolecular.org (10.08.2022).
